# Long-Term Culture Captures Injury-Repair Cycles of Colonic Stem Cells

**DOI:** 10.1016/j.cell.2019.10.015

**Published:** 2019-11-14

**Authors:** Yi Wang, I-Ling Chiang, Takahiro E. Ohara, Satoru Fujii, Jiye Cheng, Brian D. Muegge, Aaron Ver Heul, Nathan D. Han, Qiuhe Lu, Shanshan Xiong, Feidi Chen, Chin-Wen Lai, Hana Janova, Renee Wu, Charles E. Whitehurst, Kelli L. VanDussen, Ta-Chiang Liu, Jeffrey I. Gordon, L. David Sibley, Thaddeus S. Stappenbeck

**Affiliations:** 1 Department of Pathology and Immunology, Washington University School of Medicine, Saint Louis, MO 63110, USA; 2 The Edison Family Center for Genome Sciences and Systems Biology, Washington University School of Medicine, Saint Louis, MO 63110, USA; 3 Department of Molecular Microbiology, Washington University School of Medicine, Saint Louis, MO 63110, USA; 4 Boehringer Ingelheim Pharmaceuticals, Immunology and Respiratory Disease Research, Ridgefield, CT 06877, USA

**Keywords:** HopX, Lgr5, stem cell, intestine, colon, colitis, Transwell, hypoxia, unfolded protein response, air-liquid interface

## Abstract

The colonic epithelium can undergo multiple rounds of damage and repair, often in response to excessive inflammation. The responsive stem cell that mediates this process is unclear, in part because of a lack of *in vitro* models that recapitulate key epithelial changes that occur *in vivo* during damage and repair. Here, we identify a Hopx^+^ colitis-associated regenerative stem cell (CARSC) population that functionally contributes to mucosal repair in mouse models of colitis. Hopx^+^ CARSCs, enriched for fetal-like markers, transiently arose from hypertrophic crypts known to facilitate regeneration. Importantly, we established a long-term, self-organizing two-dimensional (2D) epithelial monolayer system to model the regenerative properties and responses of Hopx^+^ CARSCs. This system can reenact the “homeostasis-injury-regeneration” cycles of epithelial alterations that occur *in vivo*. Using this system, we found that hypoxia and endoplasmic reticulum stress, insults commonly present in inflammatory bowel diseases, mediated the cyclic switch of cellular status in this process.

## Highlights

• Identify a regenerative stem cell population in colitis-associated regeneration
• A long-term 2D culture to reenact the crypt homeostasis-injury-regeneration cycles
• Oxygen tension serves as a switch in injury and regeneration *in vivo* and *in vitro*

## Graphical Abstract

**Figure:**
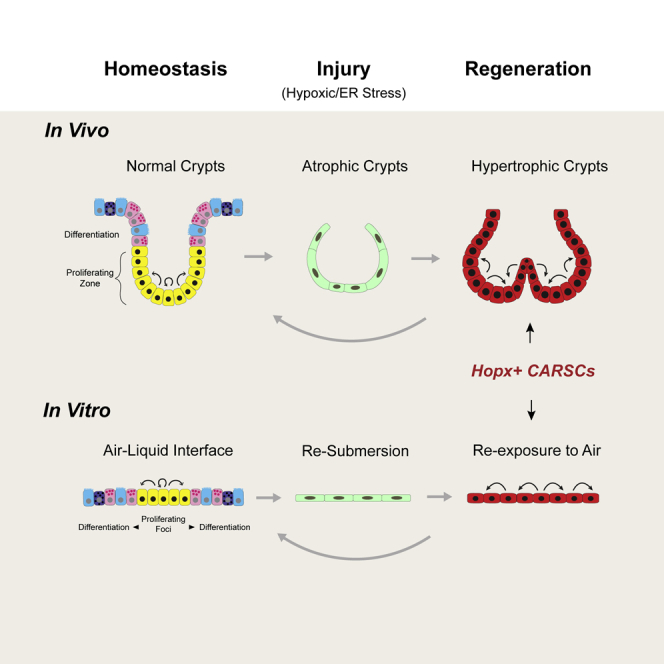

## Teaser

An Lgr5^+^-independent population of stem cells maintains homeostasis in the colon and is associated with colitis-associated regeneration.

## Introduction

The intestinal epithelium is the physical barrier separating luminal contents from the underlying stroma. Disruptions of this barrier, whether caused by infection, excessive dysregulated inflammation, vascular insults, or iatrogenic causes must be swiftly repaired to minimize the exposure of the host to insults arising from otherwise contained luminal contents.

The intestinal epithelium exploits distinct repair strategies in response to acute injuries with different degrees of severity. Insults that superficially damage the mucosa and focally remove differentiated epithelial cells are quickly repaired by the migration of adjacent epithelial cells (Feil et al., 1989, Lacy, 1988). This restitution normally occurs within minutes to hours (Feil et al., 1989, Lacy, 1987). In scenarios where more severe lesions occur that include crypt loss, stem cells in the crypts positioned adjacent to the area of injury are mobilized to repair the damage by forming wound channels (Miyoshi et al., 2012, Seno et al., 2009).

Some injuries are chronic and can involve repeated damage to crypt structures resulting in abnormal epithelial regeneration. Such aberrant mucosal healing frequently occurs in patients with inflammatory bowel diseases (IBD), and poses a challenge for achieving long-term remission (Baert et al., 2010, Henderson et al., 2011, Schnitzler et al., 2009). A key to understanding epithelial repair in response to chronic injury, such as occurs in the setting of inflammation, is to identify the stem cell populations that execute the regenerative process. Two major types of intestinal stem cells have been shown to maintain epithelial turnover in homeostasis: the fast-cycling crypt base columnar cells marked by Lgr5 (Barker et al., 2007) and a suite of slow-cycling +4 stem cells markers including Hopx, Lrig1, Tert, and Bmi1 (Breault et al., 2008, Montgomery et al., 2011, Powell et al., 2012, Sangiorgi and Capecchi, 2008, Takeda et al., 2011, Yan et al., 2012). In the mouse small intestine, Lgr5^+^ stem cells are required for regeneration in a radiation injury model (Metcalfe et al., 2014). In contrast, during colonic inflammation, they are dispensable for repair (Metcalfe et al., 2014), raising a question as to the identity of colonic stem cells mediating colitis-associated regeneration. Recent studies report a fetal-like reversion of the regenerative epithelium in both dextran sodium sulfate (DSS)-induced colitis and helminth infection (Nusse et al., 2018, Yui et al., 2018). Nevertheless, due to the lack of lineage tracing and cell ablation experiments, it is unclear whether cells in such a primitive state are functionally required for epithelial regeneration in colitis and can truly represent a regenerative stem cell population.

The complex cellular composition/architecture of the intestine poses a formidable challenge when striving to obtain a detailed, comprehensive mechanistic dissection of chronic injury and repair of its epithelium. Current 3D epithelial culture systems have limitations in their capability to model multiple cycles of injury and repair. Organoids and spheroid cultures mimic components of homeostasis (Miyoshi and Stappenbeck, 2013, Ootani et al., 2009, Sato et al., 2009, Sato et al., 2011, Spence et al., 2011). Manipulations of organoids or spheroids to induce relatively pure select lineages representing tissue homeostasis or injury are usually confounded by the short-lived nature of these cells and cellular states in culture (Basak et al., 2017, de Lau et al., 2012, Miyoshi et al., 2017, Yin et al., 2014, Yui et al., 2018). However, in pathological conditions like IBD, epithelial cells are often the subject of recurrent insults including hypoxic injury and endoplasmic reticulum (ER) stress (Giatromanolaki et al., 2003, Hatoum et al., 2005, Heazlewood et al., 2008, Kaser et al., 2008, Shkoda et al., 2007, Taylor and Colgan, 2007). As a result, intestinal epithelial cells can undergo repeated cycles of injury-repair (Sturm and Dignass, 2008), switching constantly from one cellular state to another. No *in vitro* epithelial model system has been able to recapitulate this complex process. The development of such a system would allow a better understanding of stem cell behavior during injury and subsequent regeneration and provide opportunities for creating new therapeutics.

In this report, we present the identification of a colitis-associated regenerative stem cell (CARSC) population marked by Hopx expression in mouse models of colitis. We demonstrate that Hopx^+^ CARSCs arise during the reparative stage of colitis, preceded by an injury phase when Lgr5/Hopx double negative atrophic crypts are prevalent near areas of ulcerations. Hopx^+^ CARSCs largely co-express fetal-like markers and can functionally contribute to regeneration as demonstrated by lineage tracing and cell ablation experiments. Importantly, we establish a long-term 2D colonic *in vitro* system capable of modeling Hopx^+^ CARSCs and the repeated cycles of colonic epithelial injury-regeneration. By exposing the apical side of the monolayer layer to air, *in vitro* Hopx^+^ CARSCs undergo a proliferative burst before regenerating into a self-organizing monolayer that mimics cells in homeostasis. This mature monolayer can then be re-submerged to elicit a profound and rapid damage response mimicking *in vivo* epithelial injury. Hypoxia and ER stress, insults commonly present in IBD patients and mouse models of colitis, mediate this process. Importantly the cycle of injury and repair can be completed in this model system, due to the fact the same monolayer can be re-exposed to air-liquid interface thus returning cells to a homeostatic state.

## Results

### Hopx^+^ CARSCs Promote Colitis-Associated Regeneration *In Vivo*

**Figure 1:**
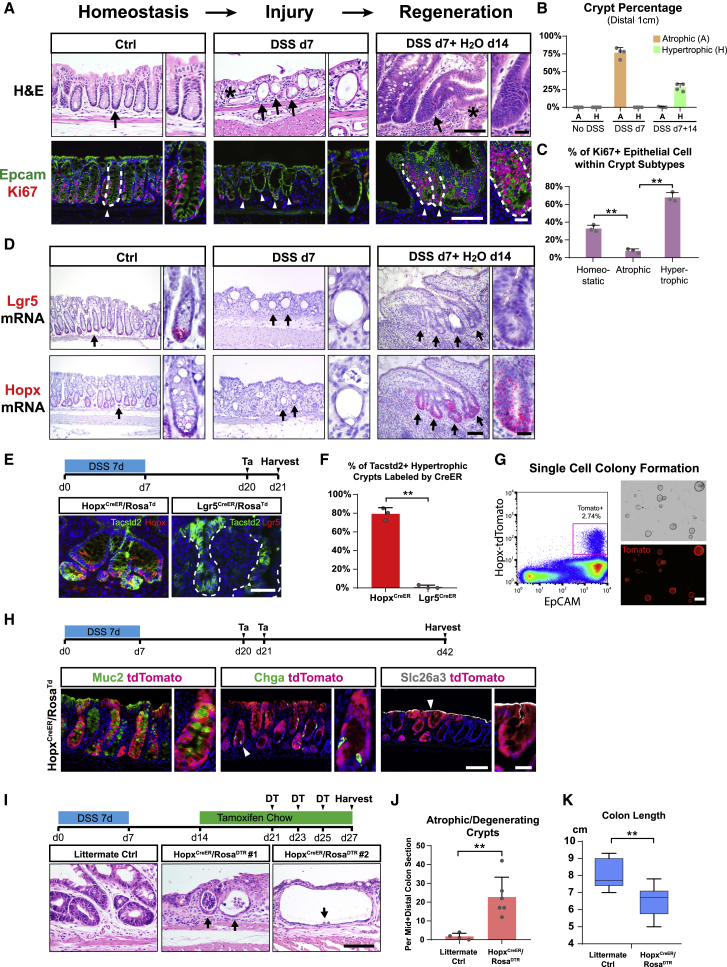
Hopx^+^ CARSCs Promote Colitis-Associated Regeneration *In Vivo* (A–D) Sections of colonic mucosa from wild-type (WT) mice treated with vehicle, 7 days of DSS, or 7 days of DSS with a 14-day “washout” period were stained with H&E (A, top panels), Epcam (green), Ki67 (red) (A, bottom panels), or *in situ* probes against Lgr5 (D, top panels) and Hopx mRNAs (D, bottom panels). Arrows and arrowheads denote crypt bases. White dashed lines indicate crypt/lamina propria boundaries. The asterisk denotes an ulcer. Percentage of atrophic (yellow) and hypertrophic (green) crypts within the distal-most colon (1 cm) under various conditions of DSS-induced colitis were plotted as mean ± SD (B) (A, atrophic crypts; H, hypertrophic crypts). The percentage of Ki67^+^ crypt epithelial cells was plotted as mean ± SD for homeostatic, atrophic, and hypertrophic crypts (C). n = 3–4 mice/group. (E and F) Transiently lineage-labeled cells (red) from *Hopx*^*CreERT2*^*/Rosa*^*Td*^ or *Lgr5*^*CreERT2*^*/Rosa*^*Td*^ mice were co-stained with Tacstd2 (green) (E). The percentage of Tacstd2^+^ crypts in the mid and distal colon that were co-labeled with tdTomato from the two CreERT2 lines was plotted as mean ± SD (F). n = 3 mice/group. (G) Single Hopx^+^ cells at the regenerative stage of DSS-induced colitis were sorted and cultured in Matrigel with 50% L-WRN media (left panel). Light and tdTomato fluorescent images of spheroids on day 6 after plating (right panels). (H) Experimental scheme for lineage tracing assays of Hopx^+^ CARSCs from *Hopx*^*CreERT2*^*/Rosa*^*Td*^ mice at the regenerative stage of DSS-induced colitis (top panel). TdTomato^+^ traced clones in the distal colon were co-stained with Muc2 (goblet cells), Chga (enteroendocrine cells), and Slc26a3 (colonocytes). (I–K) Experimental scheme for Hopx^+^ CARSCs ablation (I, top panel). Crypt morphology was defined by H&E stained colonic sections from *Hopx*^*CreER*^*/Rosa*^*DTR*^ mice (arrows in I) and their WT littermate controls subjected to the same procedure. The number of atrophic/degenerating crypts per mid + distal colon section was plotted as mean ± SD (J). n = 4–6 mice/group. Colon length was plotted as a box-whisker plot (K). n = 7–9 mice/group. Two-tailed Student’s t test in (C), (F), and (J): ^∗∗^p < 0.01. Two-tailed Mann-Whitney U test in (K): ^∗∗^p < 0.01. Bars: (G) 500 μm; (A, D, E, H, and I) 100 μm; (insets of A, D, and H) 25 μm. Histological, *in situ* hybridization and immuno-fluorescent images are representative of at least 3 mice examined. See also Figure S1.

**Figure S1:**
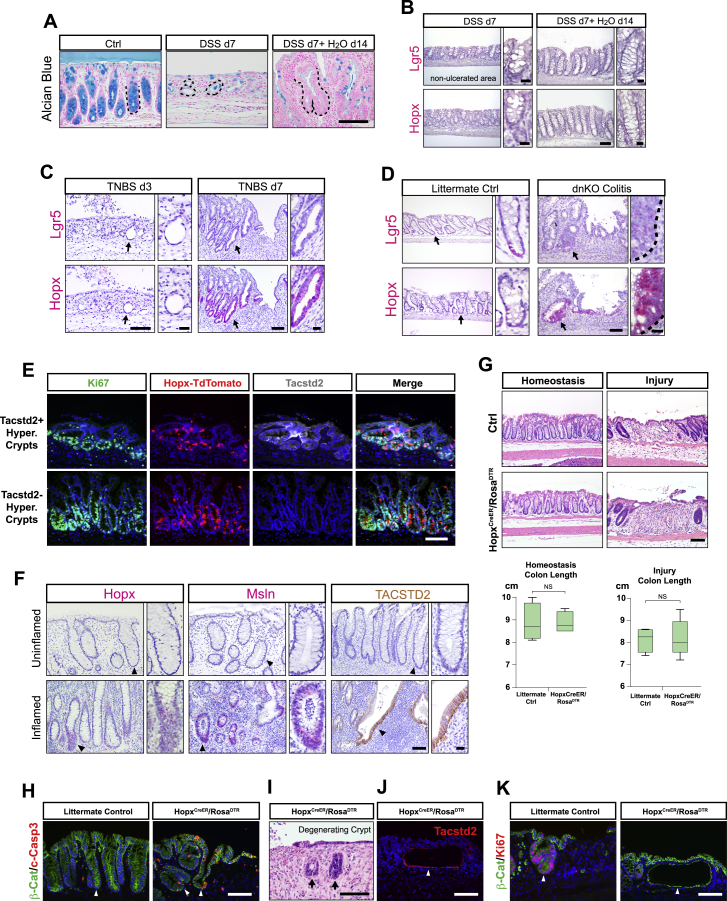
Hopx^+^ CARSCs Promote Colitis-Associated Regeneration *In Vivo*, Related to Figure 1 (A) Alcian blue staining of goblet cells present in colonic mucosa sections obtained from mice treated with no DSS, 7 days of DSS, and 7 days of DSS plus a 14-day recovery phase. Black dashed lines outlined the boundary between crypts and lamina propria. (B) *In situ* hybridization assay of Lgr5 and Hopx mRNA expression in crypts from areas with no ulcerations during the injury (DSS d7) and regenerative (DSS d7+ H_2_O d14) stages of DSS-induced colitis. Black dashed lines outlined the boundary between crypts and lamina propria. (C) *In situ* hybridization assay of Lgr5 and Hopx mRNA expression on colonic sections derived from BALB/c mice 3 and 7 days following acute injury by TNBS enema. Note the presence of abundant atrophic crypts on TNBS day 3 (arrows in left panels) and hypertrophic crypts on TNBS day 7 (arrows in right panels). The insets magnify areas indicated by the arrows. (D) Lgr5 and Hopx mRNA levels assayed by *in situ* hybridization in the dnKO spontaneous colitis model. Arrow denotes homeostatic crypts from WT littermate controls (left panels) or hypertrophic crypts from dnKO mice (right panels). The insets magnify areas indicated by the arrows. (E) Hopx+ CARSCs, transiently labeled by tamoxifen administration at the regenerative stage of DSS-induced colitis, were co-stained with Ki67 (green) and Tacstd2 (gray). Hopx-labeled hypertrophic crypts were assessed for co-localization with Tacstd2. (F) Hopx and Msln mRNA levels and TACSTD2 protein level were assayed on surgical resection or biopsy samples derived from patients with ulcerative colitis. Paired uninflamed and inflamed areas of the same sample were assayed for comparison. Images were representative of staining in the 7 patient samples. (G) H&E staining of colon sections derived from *Hopx*^*CreER*^*/Rosa*^*DTR*^ and littermate control mice that were subject to Hopx+ cell ablation during homeostasis or at the injury phase of DSS-induced colitis. Colon lengths from each group were measured and plotted as a box-whisker plot. n = 3 mice/group. (H) Epithelial apoptosis identified by co-staining of cleaved-Caspase 3 (red) and β-catenin (green) in *Hopx*^*CreER*^*/Rosa*^*Td*^ (arrow in right panel) and wild-type littermate control mice (arrow in left panel) after a single dose of diphtheria toxin. (I) Degenerating crypts (arrows) shown by H&E staining after ablation of Hopx+ CARSCs. (J) Atrophic-appearing crypts resulted from Hopx+ CARSCs ablation stained for Tacstd2 to examine co-localization (arrowhead). (K) Atrophic-appearing crypts resulted from Hopx+ CARSCs ablation were stained for Ki67 to examine cell proliferation (arrow in right panel). This was compared to the proliferation level of hypertrophic crypts in littermate controls subjected to the same procedure (arrow in left panel). Two-tailed Mann-Whitney U test in G: NS = Not Significant. Bars: (A-K) 100 μm; (insets of B, C, D and F) 25 μm. Histological, *in situ* hybridization and immuno-fluorescent images are representative of at least 3 animals examined.

DSS administration to mice typically induces damage and ulceration of the colonic mucosa. We examined crypt morphology in DSS-induced injuries with or without a recovery phase. After a 14-day recovery from DSS injury, we confirmed previous studies that showed the presence of hypertrophic crypts in the distal colon (Erben et al., 2014, Yui et al., 2018). These elongated crypts contained an increased number of proliferative cells at the expense of cellular differentiation as demonstrated by a substantial reduction in mature goblet cells (Figures 1A–1C and S1A) The close proximity of hypertrophic crypts to the areas of ulceration reflected an ongoing process of crypt regeneration, as described previously (Yui et al., 2018). In contrast, during the injury phase (with continuous DSS administration for up to 7 days), many crypts, frequently located near or within ulcerated areas in the distal colon, exhibited an atrophic appearance. These atrophic crypts contained epithelial cells with a squamous morphology instead of the columnar shape observed in untreated mice (Figure 1A); they also manifested greatly reduced proliferative activity and mature goblet cell differentiation (Figures 1A, 1C, and S1A). Atrophic crypts with these characteristics were prominent at the injury stage but were rare during regeneration (Figure 1B). Thus, atrophic crypts appeared to precede emergence of hypertrophic crypts and potentially represent an injured cellular state occurring in response to DSS injury.

We next examined the mRNA expression of markers for fast-(Lgr5) and slow-cycling (Hopx) stem cells by *in situ* hybridization at distinct stages of DSS-induced colitis. Mice administered with 7 days of DSS showed greatly decreased expression of both Lgr5 and Hopx mRNAs in both uninjured (Figure S1B) and injured areas, the latter including atrophic crypts (Figure 1D). In mice with DSS injury followed by 14 days of recovery, Lgr5 expression was still largely suppressed in most colonic crypts, including uninjured areas and regenerating hypertrophic crypts (Figures 1D and S1B). This latter finding is consistent with previously reported transcriptomic data (Yui et al., 2018). In contrast, Hopx expression re-emerged during the regeneration phase with robust expression specifically in epithelial cells located in hypertrophic crypts (Figures 1D and S1B). These data suggest that Hopx can potentially mark an arising regenerative stem cell population. A similar temporal pattern of crypt morphological changes during injury/repair was observed in response to 2,4,6-trinitrobenzene sulfonic acid (TNBS)-induced colitis (Figure S1C). As in the DSS model, neither Hopx nor Lgr5 mRNA was detected in atrophic crypts (present on day 3 post injury); in contrast, Hopx but not Lgr5 mRNA was enriched in hypertrophic crypts (present on day 7 post injury). We also observed similar findings using a genetic model of spontaneous colitis (dnKO) where hypertrophic crypts are a prominent feature (Figure S1D; Kang et al., 2008). Thus, Hopx mRNA is enriched in hypertrophic crypts in multiple models of colonic damage secondary to inflammation.

The re-emergence of Hopx expression was similar in timing and location to the induction of fetal-like markers, such as Tacstd2 (Trop2), during epithelial regeneration (Yui et al., 2018). We utilized the *Hopx-CreERT2/Rosa-LSL-tdTomato* (*Hopx*^*CreER*^*/Rosa*^*Td*^) as well as *Lgr5-EGFP-IRES-CreERT2/Rosa-LSL-tdTomato* (*Lgr5*^*CreER*^*/Rosa*^*Td*^) mice to transiently label Hopx^+^ and Lgr5^+^ cells, respectively, by tamoxifen induction during the hypertrophic crypt stage (Figure 1E). Over 70% of Tacstd2 (Trop2)^+^ crypts were labeled by the Hopx reporter, whereas <2% were labeled by the Lgr5 reporter (Figures 1E and 1F). With the caveat that possible unequal mosaicism is associated with the two reporter mouse lines, these data further support the conclusion that Hopx, but not Lgr5, mark a regenerative stem cell population within hypertrophic crypts. Interestingly, we also noted Hopx-labeling of hypertrophic crypts that were Tacstd2-negative (Figure S1E), implying heterogeneity in the composition and stages of regenerative crypts.

We next performed lineage-tracing experiments to test if Hopx marks regenerative stem cells that can contribute to the restoration of epithelial homeostasis. We labeled the Hopx-expressing cells in *Hopx*^*CreER*^*/Rosa*^*Td*^ mice after 14 days of recovery and observed tdTomato-traced clones 21 days after labeling. We found numerous tdTomato^+^ ribbons within the distal colon, particularly the most distal 1-cm segment where DSS-induced injury and regenerative events were prevalent (Figure 1H). These tdTomato-traced clones also contained multiple differentiated cell lineages including goblet cell, enteroendocrine cell, and colonocytes (Figure 1H), suggesting that the Hopx^+^ cells within hypertrophic crypts can re-establish homeostasis and possess regenerative stem cell properties. To further demonstrate the stem cell properties of these cells, we sorted single Hopx^+^ cells from colons during the regenerative stage and cultured them in Matrigel/L-WRN media. We found 1.9% ± 0.3% of these cells (n = 3 experiments) formed spheroids (Figure 1G), comparable to previously reported colony formation efficiency of singly sorted stem cells isolated from the homeostatic colon (Sasaki et al., 2016). Based on these data, we termed this cell population Hopx^+^ CARSCs.

We then tested if an equivalently marked regenerative stem cell population existed during crypt regeneration in humans. Using colonic sections from areas of active and inactive disease in ulcerative colitis subjects, we found expression of Hopx, as well as regenerative epithelial markers Tacstd2 and Msln (Gregorieff et al., 2015), were detected in hypertrophic crypts within the inflamed areas but not in uninflamed areas (Figure S1F), suggesting a similar paradigm of regeneration may occur in human colon.

To evaluate if the emerging Hopx^+^ CARSCs play a functional role in colitis-associated crypt regeneration, we ablated this population in the *Hopx*^*CreER*^*/Rosa*^*DTR*^ line at the hypertrophic stage by diphtheria toxin (DT) injection beginning at 14 days post DSS injury (Figure 1I). Elevated epithelial apoptosis was observed in the hypertrophic crypts after one dose of DT (Figure S1H). After three doses of DT, we observed a decrease in colon length and substantial increase in atrophic and degenerative appearing crypts that were rare at this stage of regeneration in littermate controls subjected to the same procedure (Figures 1I–1K and S1I). The atrophic-appearing crypts largely co-labeled with Tacstd2 (Figure S1J), implying cell ablation had occurred as intended within hypertrophic crypts. Importantly, these atrophic appearing crypts exhibited diminished proliferation and occasional dilation (Figures 1I and S1K), suggesting a disruption of normal regenerative processes driven by hypertrophic crypts. No effects on crypt morphology or colon length were detected when ablation was performed prior to or immediately after injury (Figure S1G). Together, these findings establish an essential function of the emerging Hopx^+^ CARSCs in promoting crypt regeneration in the setting of colitis.

### *In Vitro* Culture of a Self-Organizing Colonic Epithelial Monolayer

**Figure 2:**
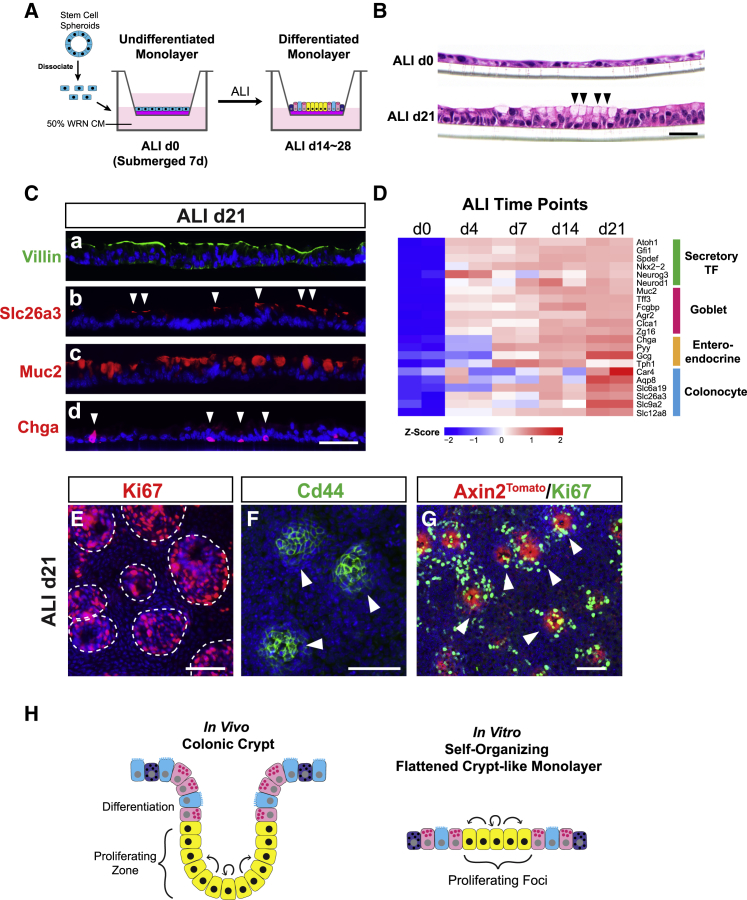
*In Vitro* Culture of a Self-Organizing Colonic Epithelial Monolayer (A) Experimental scheme for ALI culture. (B) H&E stained sections of Transwell monolayers showed morphological changes from ALId0 to ALId21 including signs of goblet cell differentiation on ALId21 (arrowheads). (C) Sections of ALId21 monolayers stained for Villin (brush border, a), Slc26a3 (colonocytes, b, arrowheads), Muc2 (goblet cells, c), and Chga (enteroendocrine cells, d, arrowheads). (D) Heatmap of secretory cell transcription factors as well as goblet, enteroendocrine, and colonocyte markers at various stages of ALI culture. n = 2 replicates/time point. (E and F) Whole mount staining for Ki67 (E) and CD44 (F) on ALId21 marked numerous self-organizing proliferative foci. (G) ALI monolayers derived from *Axin2*^*tdTomato*^ reporter line on ALId21 were co-stained for RFP (red) and Ki67 (green, arrowheads). (H) Diagram demonstrating that mature *in vitro* ALI cultures resemble flattened crypts *in vivo*. Bars: (B) 25 μm; (C and E–G) 50 μm. Histological and immuno-fluorescent images are representative of at least 3 Transwell samples examined. See also Figure S2 and Tables S1 and S2.

**Figure S2:**
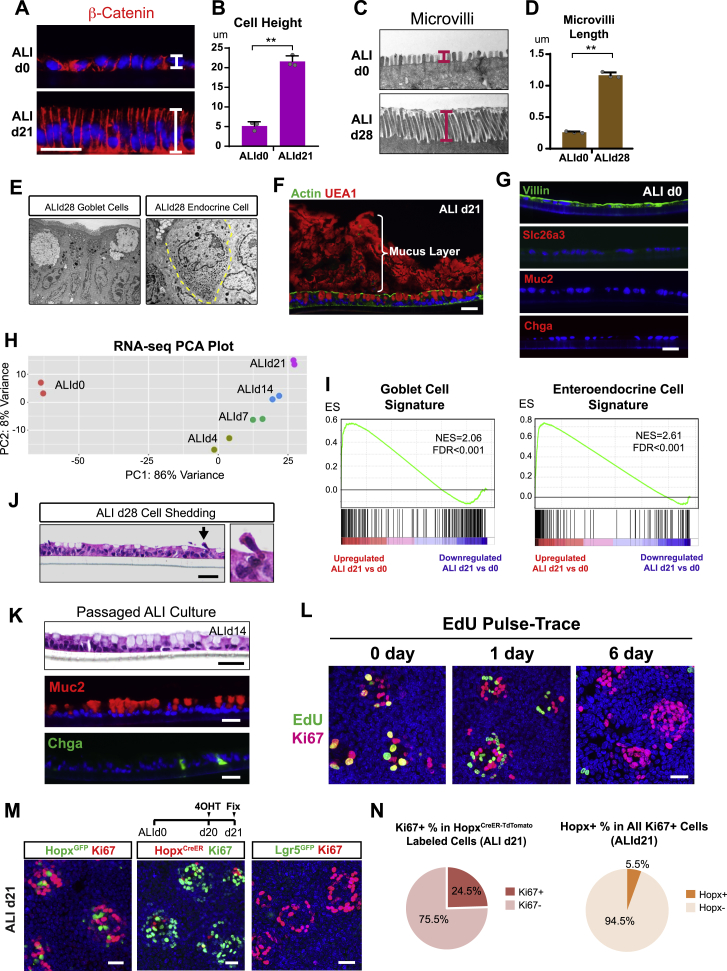
*In Vitro* Culture of a Self-Organizing Colonic Epithelial Monolayer, Related to Figure 2 (A-B) β-catenin staining demonstrated the distinct cell shapes on ALId0 and ALId21 (A). Cell height was quantified and plotted to compare ALId21 and ALId0 (B). n = 3 samples/group. (C-D) Microvilli length measured in the transmission electron microscopy images and compared between ALId28 and ALId0. n = 3 samples/group (10 cells per sample) (E) Transmission electron microscopy images showed the morphology of goblet cells (left panel) and enteroendocrine cells with secretory granules (right panel). Yellow dashed line outlined an enteroendocrine cell. (F) Actin (green) and UEA1 (red) double staining described polarization and the presence of a mucus layer in ALId21 monolayers. (G) Immunostaining of major colonic epithelium lineage markers on ALId0 monolayers. (H) Principal component analysis of transcriptomic profiles of monolayers harvested on ALId0, ALId4, ALId7, ALId14 and ALId21. (I) GSEA-based analysis comparing the mRNA profiles of ALId21 cells to published goblet (left panel) and enteroendocrine cells (right panel) (Jadhav et al., 2017). See also Tables S1 and S2. (J) H&E staining showed a cell extrusion event (arrow and higher power view on the right) on ALId21. (K) ALId21 monolayer culture was dissociated by trypsin digestion and re-plated onto a new Transwell followed by 7 days of submersion and 14 days of ALI. Cell morphology after the passaging procedure is defined by H&E staining. Goblet cell and enteroendocrine cell differentiation was demonstrated by Muc2 and Chga staining. (L) EdU and Ki67 co-staining on monolayers that were labeled by EdU incorporation for 3 h and washed out for various times (0, 1 and 6 days). (M-N) Hopx^GFP^+ (left panel in M), Lgr5^GFP^+ (right panel in M) or Hopx^CreERT2^ (middle panel in M) transiently labeled cells co-stained for Ki67 on ALId21. The proliferation rate of Hopx-labeled cells on ALId21 was plotted in the form of a pie chart (left panel in N). The percentage of Hopx+ cells within the Ki67% loci was plotted as a pie chart (right panel in N). n = 3 samples/group. Mean values ± SD are shown. Two-tailed Student’s t test: ^∗^p < 0.05; ^∗∗^p < 0.01. Bars: (A, F, G, J, K, L and M) 25 μm. Histological, *in situ* hybridization, EM and immuno-fluorescent images are representative of at least three samples examined.

As we demonstrated above, the dynamic nature of crypt remodeling and the power of epithelial regeneration are remarkable in colitis, but the extent to which these processes are epithelial intrinsic is unclear. Deconstructing the epithelial-specific capacity calls for an *in vitro* system that permits colonic epithelial cells to seamlessly switch between multiple cellular states reflecting distinct stages of *in vivo* regeneration. A first step toward this goal was to establish a long-term *in vitro* culture that contains features of colonic crypt epithelial cells in steady state. To this end, we adopted and modified a Transwell method (Wang et al., 2015) to culture adult mouse colonic epithelial cells in two dimensions. Colonic spheroids were dissociated into single cells (Miyoshi and Stappenbeck, 2013, Moon et al., 2014) and plated onto Transwell membranes. Cells were exposed to air-liquid interface (ALI) for 14 to 28 days after being submerged in L-WRN conditioned medium for 7 days (Figure 2A). Surprisingly, we did not observe crypt-like folding structures as previously reported (Wang et al., 2015), but rather a continuous 2D monolayer of epithelium. The presence of a feeder layer of irradiated 3T3 cells did not alter the 2D monolayer morphology (data not shown). Interestingly, cells at day 0 and day 21 after exposure to the air-liquid interface (ALId0, ALId21) showed drastically different cell morphologies. H&E stained sections of ALId0 cells revealed a squamous morphology with no indication of cellular differentiation, while ALId21 cells were columnar and included a population with goblet cell features (Figure 2B). β-catenin staining of monolayer sections demonstrated that the cell height on ALId21 was similar to *in vivo* colonic crypts (Figures S2A and S2B). In addition, microvilli length on ALId28 measured ∼1 μm, comparable to the microvillar length of absorptive cells *in vivo* (Figures S2C and S2D).

We used several approaches to define the extent to which differentiated cell lineages were induced by the switch to ALI. Immunostaining and transmission electron microscopic images demonstrated that the ALId21 colonic monolayers contained all major differentiated cell types including goblet cells (marked by Muc2), enteroendocrine cells (marked by Chga), and absorptive colonocytes (marked by Slc26a3) (Figures 2C and S2E). Goblet cells were capable of secreting mucus that overlaid the epithelium (Figure S2F). The percentages of goblet and enteroendocrine cells were 34.5% ± 4.8% and 1.9% ± 0.4%, respectively (n = 3 experiments). The allocation of these two lineages was slightly higher than *in vivo* (Tóth et al., 2017), although the number could vary among different segments of the colon. In contrast, no cellular differentiation was detectable in ALId0 cells (Figure S2G). RNA sequencing (RNA-seq) analysis of epithelial monolayers at multiple time points after the switch to ALI, including ALId0, ALId4, ALId7, ALId14, and ALId21, showed a clear evolution of RNA profiles over time (Figure S2H). Expression of transcription factors crucial for specification of secretory lineage differentiation, including the master regulator Atoh1, were first detected on as early as ALId4 (Figure 2D). The goblet cell transcriptional signature emerged by ALId4 and was followed by a substantial upregulation of enteroendocrine cell markers on ALId7 (Figure 2D). Colonocyte markers increased later between ALId14 and ALId21 (Figure 2D). We next utilized gene set enrichment analysis (GSEA) to compare the transcriptomic profile of ALId21 monolayers to those derived from sorted goblet cells and enteroendocrine cells *in vivo* (Jadhav et al., 2017). This revealed a significant enrichment of goblet and enteroendocrine cell signatures in the ALId21 monolayers (Figure S2I; Tables S1 and S2). These findings depict a dynamic stepwise lineage differentiation process after the ALI switch and indicate d21 ALI culture comprises the major epithelial cell lineages found in a homeostatic crypt.

The long-term feature of ALI culture together with apparent cell shedding and turnover (Figure S2J) suggested the presence of a stable, self-renewal component to the monolayers. Supporting this idea, we were able to passage ALId21 cells and re-establish a new mature monolayer (Figure S2K). Remarkably, whole mount staining of ALId21 cells revealed numerous foci of proliferative cells positive for both Ki67 and Cd44 (Figures 2E and 2F), biomarkers of crypt base cells *in vivo*. A widely held notion is that a Wnt ligand gradient is crucial for formation of the proliferative compartment at the crypt base (Clevers, 2013, Farin et al., 2016); however, cells in the ALI culture were all exposed to the same level of Wnt-containing medium and lacks the Wnt-producing Paneth cells reported to shape the gradient that creates the organization of small intestinal organoids (Farin et al., 2016, Sato et al., 2011). We found that Axin2, a target of canonical Wnt signaling, is primarily co-localized to the Ki67^+^ foci (Figure 2G), demonstrating a differential response to Wnt exists in the absence of a ligand gradient in ALI culture.

Consistent with active cellular turnover, ethynyldeoxyuridine (EdU) pulse-chase experiments showed that the transiently labeled proliferative cells (EdU^+^) had largely exited proliferative foci by day 6 post labeling. This timing was similar to *in vivo* mouse colons (Figure S2L; Karam, 1999). Furthermore, Ki67^+^ foci harbored Hopx but not Lgr5-expressing cells (Figure S2M); 25% ± 6% of these Hopx^+^ cells were in a proliferative state (Figure S2N), a rate similar to the slow-cycling +4 intestinal epithelial stem cells *in vivo* (Bankaitis et al., 2018, Li et al., 2016, Montgomery et al., 2011, Powell et al., 2012). Within the Ki67^+^ foci, 5.5% ± 0.3% of cells were Hopx^+^ (Figure S2N). These findings suggested that the *in vitro* homeostasis-like status of ALId21 is fueled by Hopx-expressing “+4-like” stem cells, confirming the dispensability of Lgr5^+^ stem cells for homeostasis (Tian et al., 2011). We speculate that certain components in the L-WRN media may have favored such stem cell choices. Moreover, the self-organizing nature of the mature monolayer in Transwell ALI culture can be utilized to successfully support the complete sexual growth cycles of Cryptosporidium (Wilke et al., 2019), a challenging epithelial parasite to grow *in vitro*. Taken together, we have established a unique 2D sheet of colonic epithelial cells that self-organizes in the absence of a Wnt gradient and mimics “flattened” homeostatic crypts *in vivo* (Figure 2H).

### *In Vitro* Modeling of Hopx^+^ CARSCs

**Figure 3:**
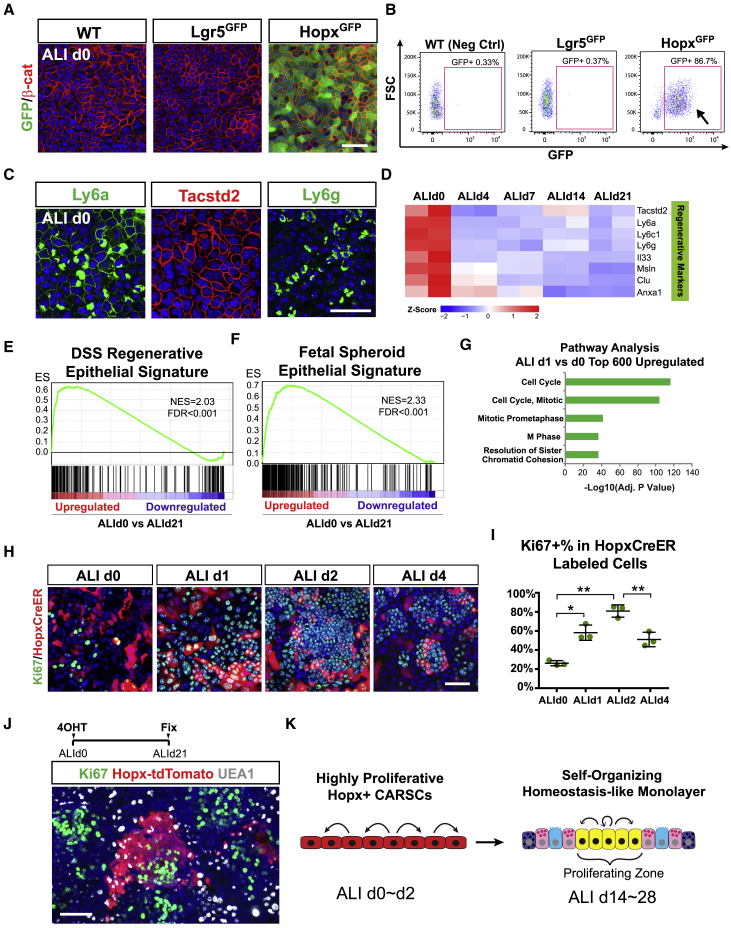
*In Vitro* Modeling of Hopx^+^ CARSCs (A and B) Whole mount images of ALId0 monolayers from WT, *Lgr5*^*GFP*^, and *Hopx*^*GFP*^ mice assessed for GFP (green) expression and co-stained for β-catenin (red) (A). Percentage of GFP^+^ cells in these monolayers as examined by flow cytometry (B). (C) Whole mount images of ALId0 monolayers stained for fetal markers Ly6a, Tacstd2, and Ly6g. (D) Heatmap of selected fetal markers in cells analyzed on ALId0, ALId4, ALId7, ALId14, and ALId21. Data are row normalized. n = 2 replicates/time point. (E and F) GSEA-based analysis comparing the mRNA profiles of ALId0 cells to the regenerative epithelium of DSS-treated mice (E) and fetal spheroid epithelium (F) (Mustata et al., 2013, Yui et al., 2018). Normalized enrichment score (NES) = 2.03 (E) and 2.33 (F). False discovery rate (FDR) < 0.001. See also Tables S3 and S4. (G) Pathway analysis (Enrichr) comparing the transcriptional signature of ALId1 cells to ALId0 cells. (H and I) Whole mount images for Ki67 staining (green) of *Hopx*^*CreER*^/*Rosa*^*Td*^-labeled cells (red) at early ALI time points (H). The proliferative rate of *Hopx*^*CreER*^-labeled cells at designated ALI time points was plotted as mean ± SD (I). n = 3 samples/group. (J) Co-staining of Ki67 (green) and UEA1 (gray, goblet cells) with respect to Hopx^+^ clones (red) lineage-traced from ALId0 to ALId21. (K) A diagram showing that the *in vitro* Hopx^+^ CARSCs are highly proliferative on ALId0 to ALId2 and are capable of regenerating and giving rise to a homeostasis-like mature ALI monolayer. Two-tailed Student’s t test: ^∗^p < 0.05; ^∗∗^p < 0.01. Bars: (A, C, H, and J) 50 μm. Histological, immuno-fluorescent, and flow cytometry images are representative of at least 3 Transwell samples examined. See also Figure S3.

**Figure S3:**
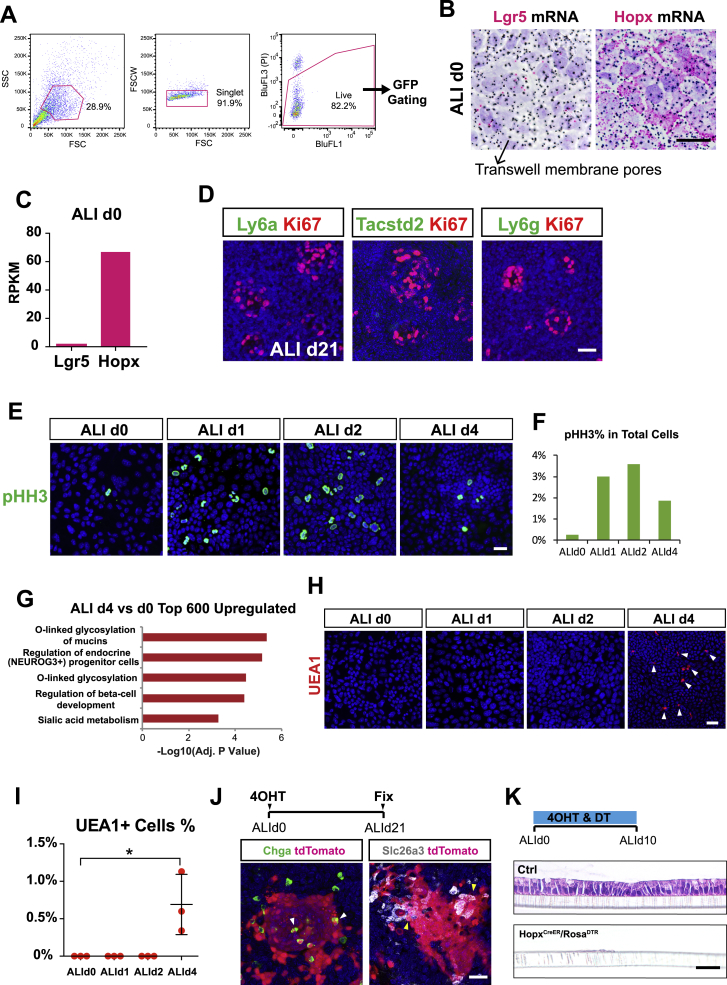
*In Vitro* Recreation of Hopx^+^ CARSCs and Its Regenerative Potential, Related to Figure 3 (A) Detailed flow cytometry gating strategies used to select live single cells for subsequent GFP analysis in WT, Lgr5^GFP^ and Hopx^GFP^ ALId0 cells as shown in Figure 3B. SSC = Side scatter. FSC = Forward scatter. BluFL1 was used to check for autofluorescence of the BluFL3 (propidium iodide, PI) channel which is a live/dead indicator. (B) Whole mount *in situ* hybridization on Transwells to assay Lgr5 and Hopx mRNA levels in ALId0 monolayers (red). Black dots represent the pores of the Transwell membrane. (C) Expression of Lgr5 and Hopx mRNAs on ALId0 as defined by RNA-Seq. (D) Whole mount Ki67 (red) co-staining with Ly6a (green, left), Tacstd2 (green, middle) and Ly6g (green, right) on ALId21 monolayers. (E-F) Whole mount phospho-histone H3 staining on Transwells examined on ALId0, ALId1, ALId2 and ALId4. The mitotic rate for these time points was plotted in panel F. (G) Top KEGG pathways upregulated in ALId4 monolayers in comparison to ALId0. (H-I) Whole mount UEA1 staining of Transwells sampled on ALId0, ALId1, ALId2 and ALId4 to characterize the time course of goblet cell differentiation. The percent of UEA1+ cells is indicated in (I). n = 3 samples/group. (J) Whole mount co-staining of Chga and Slc26a3 with tdTomato following 21 days of lineage tracing experiment on ALI cultures derived from *Hopx*^*CreER*^*/Rosa*^*Td*^ mice. (K) H&E staining of ALI culture sections after 10 days of Hopx+ cell ablation. Mean values ± SD are shown. Two-tailed Student’s t test: ^∗^p < 0.05. Quantification data are represented as mean ± SD. Bars: (B, D, E, H, J and K) 25 μm. *In situ* hybridization and immuno-fluorescent images are representative of at least three samples examined.

To address questions about how the homeostasis-like monolayer forms on ALId21 and what cells give rise to this stable monolayer, we characterized the nature of cells in the undifferentiated ALId0 epithelium. We initially hypothesized that Lgr5^+^ stem cells, a main crypt stem cell population present under conditions of homeostasis, would predominate. However, we did not observe detectable GFP signal in monolayers derived from *Lgr5*^*GFP*^ mice (Figure 3A). In contrast, monolayers derived from *Hopx*^*GFP*^ mice showed readily detectable positive signals in most cells (Figure 3A). Fluorescence-activated cell sorting (FACS) analysis of ALId0 cells from the two GFP lines confirmed the whole mount staining results (Figures 3B and S3A). *In situ* hybridization demonstrated low levels of detectable Lgr5 mRNA transcripts in contrast to Hopx (Figure S3B). Lgr5 and Hopx mRNAs levels on ALId0, defined by RNA-seq, supported the *in situ* hybridization results (Figure S3C). Taken together, these results suggest that the negative *Lgr5*^*GFP*^ signal on ALId0 was not simply due to genetic mosaicism of this mouse line.

Hopx not only labels the +4 stem cells during homeostasis (Takeda et al., 2011), but as we showed above, also marks a stem cell population arising from colitis-associated regeneration. To distinguish which of these two cell types are represented by ALId0 monolayers, we stained whole mounts of monolayers using multiple markers of regenerative epithelium, including Ly6a (Sca1), Tacstd2 (Trop2), and Ly6g (Yui et al., 2018). We found high expression of each of these markers on ALId0 (Figure 3C), which then decreased on ALId21 (Figure S3D). This finding was verified by RNA-seq analysis (Figure 3D). To further test if ALId0 cells express a broad signature mimicking the regenerative epithelium, we compared the ALId0 RNA profile to published transcriptomic datasets for regenerative epithelial cells isolated from DSS-treated mice (Yui et al., 2018). GSEA showed a highly significant enrichment of a colitis-associated regenerative epithelial signature in ALId0 compared to ALI21 cells (Figure 3E; Table S3). Similarly, ALId0 cells are also enriched for a fetal-like signature (Figure 3F; Table S4; Mustata et al., 2013), consistent with the concept of fetal-like reprograming during epithelial regeneration.

To detect cellular changes that occurred prior to ALId4, we performed RNA-seq analysis on ALId0, ALId1, ALId2, and ALId4 monolayers. Pathway analysis of the top 300 upregulated genes comparing either ALId1 or ALId2 to ALId0 revealed a robust signature of cell-cycle progression (Figure 3G and data not shown). Staining for Ki67 and phospho-histone H3 (pHH3) demonstrated a burst of proliferation within *Hopx*^*CreER*^-labeled regenerative stem cells on ALId1, which peaked on ALId2 and was reduced on ALId4 (Figures 3H, 3I, S3E, and S3F). In contrast, a comparison of ALId4 versus ALId0 transcriptomes primarily showed pathways involved in goblet and enteroendocrine cell differentiation (Figures S3G–S3I). These data suggest that the highly proliferative Hopx^+^ cells that exist between ALId0 and ALId2 resemble the *in vivo* Hopx^+^ CARSCs arising from hypertrophic crypts.

To evaluate if the *in vitro* Hopx^+^ CARSCs can functionally regenerate and give rise to the homeostasis-like monolayers, we performed lineage tracing on Hopx-labeled cells beginning from ALId0 and found that on ALId21, tdTomato^+^ clones contained both Ki67^+^ foci as well as differentiated lineages, such as goblet cells, enteroendocrine cells, and colonocytes that extended beyond the Ki67 foci (Figures 3J and S3J). Ablating the Hopx^+^ CARSCs *in vitro* starting from ALId0 completely prevented mature monolayer formation (Figure S3K). Taken together, between ALId0 and ALId2, Hopx^+^ CARSCs undergo a burst of proliferation highly reminiscent of their *in vivo* counterparts located in the hypertrophic crypts of DSS-treated mice. Subsequently, these regenerative stem cells are capable of giving rise to differentiated lineages and forming self-organizing monolayers, thereby recapitulating a key regenerative process associated with colitis (Figure 3K).

### Recapitulating Cycles of Colonic Epithelial Injury-Regeneration *In Vitro*

**Figure 4:**
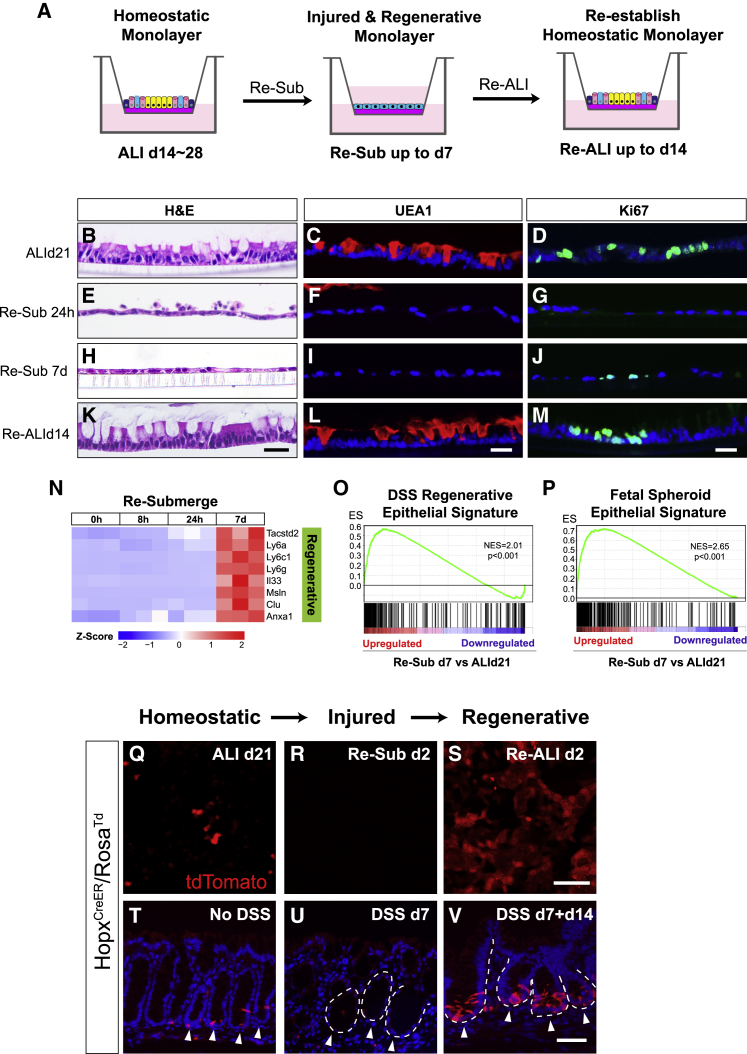
Recapitulating Cycles of Colonic Epithelial Injury-Regeneration *In Vitro* (A) Scheme of mature ALI culture re-submersion and re-exposure to air. (B–M) ALI monolayers prior to re-submersion (ALId21; B–D), 24 h (Re-Sub 24h; E–G), and 7 days (Re-Sub 7d; H–J) after re-submersion, as well as 14 days after re-exposure to ALI (Re-ALId14; K–M) were examined by H&E staining (B, E, H, and K), immunostaining for UEA1 (C, F, I, and L) and Ki67 (D, G, J, and M). (N) Heatmap of regenerative epithelial markers at 0 h, 8 h, 24 h, and 7 days after re-submersion. Data are row normalized. n = 3 replicates/time point. (O and P) GSEA-based analysis comparing the mRNA profiles of re-submerge d7 cells to the regenerating epithelium of DSS-treated mice and fetal spheroid epithelium (Mustata et al., 2013, Yui et al., 2018). NES = 2.01 (O) and 2.65 (P). FDR < 0.001. See also Tables S5 and S6. (Q–S) Whole mount images of ALI cultures derived from *Hopx*^*CreER*^*/Rosa*^*Td*^ mice. The presence of Hopx-expressing cells (red) at indicated time points (Q, ALId21; R, Re-sub d2; S, Re-ALI d2) was examined by transiently labeling monolayers with 4-OH tamoxifen 24 h before imaging. (T–V) Sections of mouse colons from *Hopx*^*CreER*^*/Rosa*^*Td*^ mice treated with no DSS, DSS for 7 days (DSS d7), and DSS for 7 days with a 14-day recovery phase (DSS d7 + d14). Hopx^+^ cells (red) were transiently labeled with tamoxifen within homeostatic (T), atrophic (U), and hypertrophic crypts (V) (arrowheads in T–V, respectively). White dashed lines mark the crypt/stroma boundary. Bars: (K–M) 25 μm; (S and V) 50 μm. Histological and immuno-stained images are representative of at least 3 Transwell samples or animals examined. See also Figure S4.

Given the impact of the switch to ALI on regenerating a mature, self-organized epithelial monolayer, we next investigated if this regenerative program can be reversed by simply re-submerging the differentiated ALI monolayer (Figure 4A), and if so, what is the timing and scope of the reversal. We found that the regenerative program that took ∼3 weeks to execute could be nearly completely reversed after 24 h of re-submersion. By this time, the epithelial cells had become squamous, lacked goblet cell phenotypes, and halted their proliferative activity (Figures 4B–4G). All these features resembled those of atrophic crypts prevalent near or within DSS-induced ulcerations. Subsequently, the monolayer re-acquired low levels of proliferation over the course of 7-day re-submersion (Figures 4H–4J). Strikingly, re-exposing this monolayer to ALI led to restoration of a columnar epithelium that consisted of both differentiated cells and proliferating compartments (Figures 4K–4M), suggesting that the re-submerged monolayer possessed regenerative potential after undergoing an atrophic crypt-like phase deprived of proliferation.

**Figure S4:**
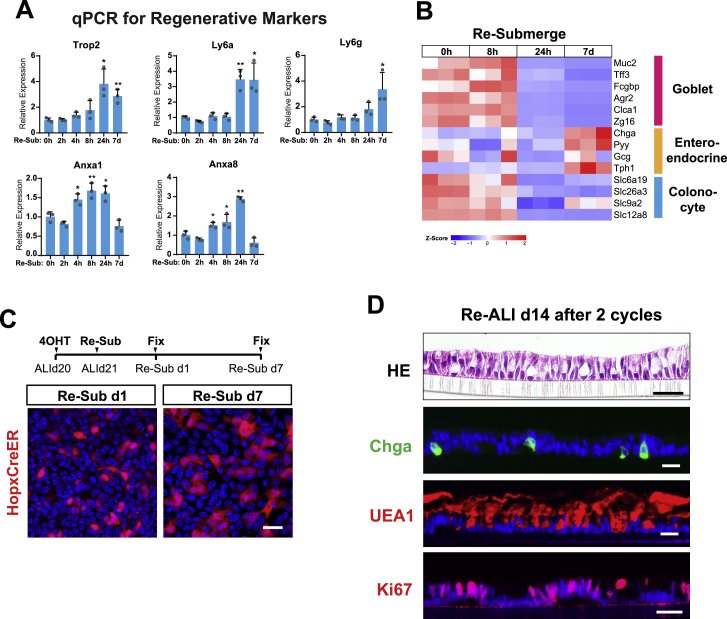
Recapitulating Cycles of Colonic Epithelial Injury Regeneration *In Vitro*, Related to Figure 4 (A) Expression of regenerative epithelial markers was analyzed by qPCR on monolayers re-submerged for 0hr, 4hrs, 8hrs, 24hrs and 7 days. Results were demonstrated by the relative expression fold changes in comparison to the 0hr time point. Student’s t test was performed to compare each individual time points after re-submersion to the 0hr time point. n = 3 samples/group. (B) Heatmap panel showing goblet cell, enteroendocrine cell, and colonocyte marker expression in monolayers re-submerged for the indicated time periods. Expression data are row normalized. (n = 3 replicates/time point). (C) ALI monolayers derived from *Hopx*^*CreER*^*/Rosa*^*Td*^ mice were transiently labeled with 4OH-Tamoxifen 1 day before re-submersion on ALId21. Labeled Hopx+ cells were traced after re-submersion for 1 day and 7 days. (D) ALI monolayers subjected to 2 cycles of re-submersion and re-ALI stained with H&E staining, and for the indicated biomarkers of major cell lineages (Chga for enteroendocrine and UEA1 for goblet cell), and proliferating cells (Ki67). Mean values ± SD are shown. Two-tailed Student's t test: ^∗^p < 0.05; ^∗∗^p < 0.01. Bars: (C and D) 25 μm. Histological and immuno-fluorescent images are representative of at least three samples examined.

Corroborating the histologic findings, RNA-seq analysis conducted at various time points after re-submersion revealed a gradual acquisition of a regenerative signature over time (Figure 4N), most notably shown by the close resemblance of the RNA profile of re-submerged day 7 cells to DSS-associated regenerative epithelium and fetal organoids (Figures 4O and 4P; Tables S5 and S6; Mustata et al., 2013, Yui et al., 2018). We then performed qPCR analysis on select regenerative markers and confirmed their increased expression over time after re-submersion (Figure S4A). Interestingly, goblet and absorptive cells (and their corresponding markers) were completely lost within the first 24 h and remained so during the 7-day re-submersion (Figure S4B). However, expression of enteroendocrine cell markers recovered over the 7 days of re-submersion (Figure S4B).

Intriguingly, transient labeling of Hopx-expressing cells revealed a temporary loss of Hopx^+^ stem cells 24 h after re-submersion (Figures 4Q and 4R). This finding was similar to the loss of Hopx expression in DSS-induced atrophic crypts (Figures 4T and 4U). However, Hopx expression re-emerged in the culture 2 days following re-exposure to air (Figure 4S), highly reminiscent of the Hopx^+^ CARSCs arising from the repair stage of DSS-induced colitis (Figure 4V). When labeled before re-submersion, at least a subset of the Hopx^+^ “+4-like” stem cells present on ALId21 survived through the re-submersion period (Figure S4C), suggesting these cells are one of the sources contributing to reprogramming *in vitro*. The recovery of the monolayer after two cycles of re-submersion and re-ALI (Figure S4D) emphasizes how this *in vitro* system can be used to mimic the cycles of colonic epithelial injury and regeneration observed *in vivo* and dissect the underlying mechanisms.

### Re-submersion Induces Cellular Stress Mediated by Low Oxygen Tension

**Figure 5:**
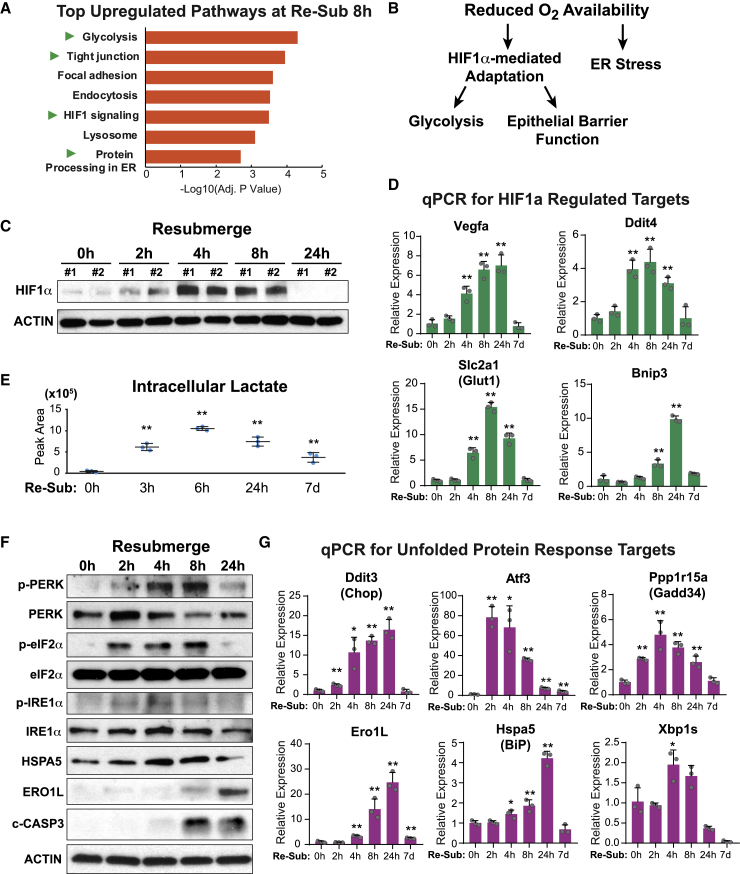
Re-submersion Induces Cellular Stresses Mediated by Low Oxygen Tension (A) Top upregulated pathways in monolayers 8 h after re-submersion as compared to ALId21. (B) A schematic summary of the cellular pathways activated by low oxygen tension. (C) Hif1α protein accumulation evaluated by immunoblot of cell lysates derived from monolayers re-submerged for 0, 2, 4, 8, and 24 h. (D) Hif1α targets expression defined by qPCR assays in monolayers re-submerged for 0, 2, 4, 8, 24 h and 7 days. Results were expressed relative to the 0 h time point. Mean ± SD are shown. n = 3 samples/time point. (E) Intracellular lactate levels in monolayers as a function of time after re-submersion. n = 3 samples/group. (F) Changes in levels of proteins known to be involved in the UPR pathway defined by immunoblotting of cell lysates as a function of time after re-submersion. (G) Expression of UPR target genes, defined by qPCR, in monolayers at different times after re-submersion. Results were expressed relative to the 0 h time point. Mean ± SD were plotted. n = 3 samples/group. Two-tailed Student’s t test relative to 0 h: ^∗^p < 0.05; ^∗∗^p < 0.01. Immunoblots are representative of two independent experiments.

To elucidate the mechanism by which the differentiated ALI monolayers changed so profoundly after re-submersion, we compared RNA-seq data generated from cells re-submerged for 8 h to those that had not been re-submerged. One of the top regulated pathways (Figure 5A), HIF1-mediated signaling, implicated the presence of hypoxic stress. In fact, several other top pathways, including glycolysis, tight junction (epithelial barrier), and protein processing in the ER (Figure 5A), can all be altered by reduced oxygen tension in either a HIF-dependent or -independent manner (Figure 5B; Glover et al., 2016, Majmundar et al., 2010, Wouters and Koritzinsky, 2008). In line with the activation of HIF1-mediated signaling, HIF1α protein accumulation, one of the hallmarks of cellular hypoxia, was observed between 2 and 8 h after re-submersion (Figure 5C). qPCR analysis confirmed a robust but transient increase of several well-known target genes directly regulated by the HIF1α transcription factor (Figure 5D). Furthermore, mass spectrometry of cell extracts showed a marked increase in intracellular lactate levels following re-submersion (Figure 5E), indicating a likely switch to glycolysis under the influence of low oxygen tension.

Low oxygen tension can also lead to ER stress (Koritzinsky et al., 2006, Koumenis et al., 2002, Ma and Hendershot, 2003, Romero-Ramirez et al., 2004). This is most likely because protein folding in the ER requires the participation of oxygen (Bader et al., 1999, Frand and Kaiser, 1999, Tu et al., 2000). ER stress is known to activate the unfolded protein response (UPR) (Oslowski and Urano, 2011). Examination of signaling cascades that constitute UPR revealed a marked elevation of both phospho-PERK and phospho-eIF2α levels within the first 8 h of re-submersion, which were subsequently diminished at 24 h (Figure 5F). IRE1α phosphorylation and Xbp1 splicing, a second arm of UPR, also showed similar dynamics after re-submersion (Figure 5F). UPR activation was further evidenced by increased protein and/or RNA levels of multiple well-known downstream target genes crucial in assisting cellular adaptations to ER stress (Figures 5F and 5G). ER stress can also lead to apoptosis, which was confirmed by the observation of elevated cleaved-Caspase 3 by 8 h after re-submersion (Figure 5F). Occurrence of ER stress also explained the rapid loss of goblet cells following re-submersion, as these cells are known to be particularly susceptible to ER stress (Heazlewood et al., 2008). More importantly, high levels of hypoxic and ER stress were reported in both IBD patients and mouse models of colitis (Giatromanolaki et al., 2003, Hatoum et al., 2005, Karhausen et al., 2004, Kaser et al., 2008, Shah et al., 2008, Shkoda et al., 2007). Together, these findings suggest that re-submersion of the culture induces acute hypoxic and ER stress, both of which are likely to act as triggers for cellular injury and profoundly alter the epithelial transcriptional profiles, metabolic pathways as well as cell fate decisions.

### Oxygen Tension Functions as an Important Switch between Injury and Regeneration

**Figure 6:**
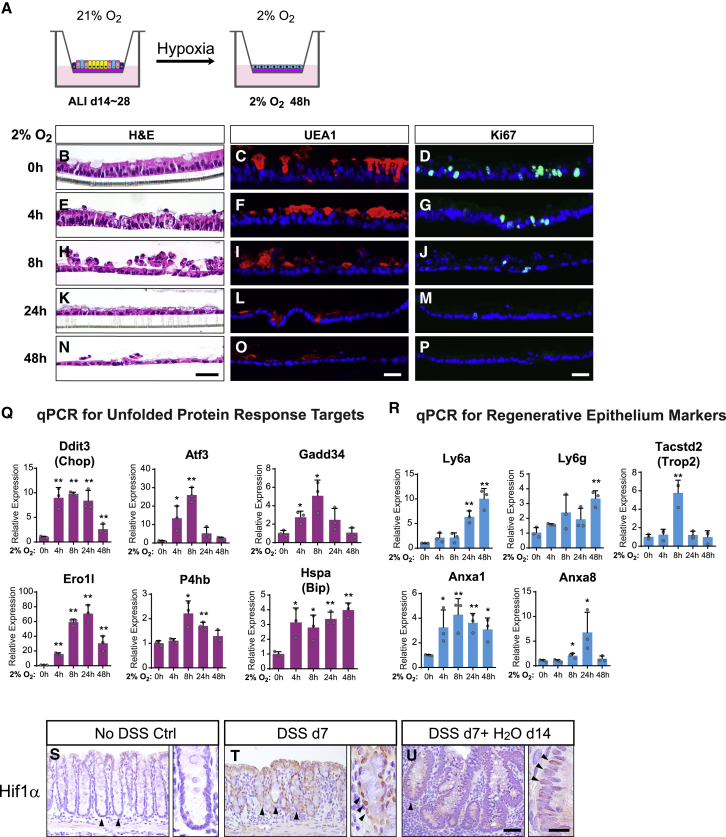
Oxygen Tension Functions as an Important Switch between Injury and Regeneration (A) Scheme of mature ALI cultures exposed to 2% O_2_ for up to 48 h while still maintaining ALI. (B–P) Staining for H&E (B, E, H, K, N), UEA1 (goblet cells; C, F, I, L, O), and Ki67 (D, G, J, M, P) on monolayers after exposed to 2% O_2_ for 0 (B–D), 4 (E–G), 8 (H–J), 24 (K–M), and 48 h (N–P). (Q and R) qPCR assays showing relative expression of UPR target genes (Q) and genes involved in epithelial regenerative responses (R) in monolayers exposed to 2% O_2_ for the indicated time periods. Results were expressed relative to the 0 h time point. Mean ± SD are shown. n = 3 samples/group. (S–U) Immunohistochemical staining of Hif1α protein on colonic mucosa sections from mice treated with no DSS, 7 days of DSS, and 7 days of DSS plus a 14-day recovery phase. Arrowheads denote the nuclear expression of Hif1α in epithelial cells of homeostatic (S), atrophic (T), and hypertrophic crypts (U). Two-tailed Student’s t test: ^∗^p < 0.05; ^∗∗^p < 0.01. Bars: (N–P) 25 μm; (U) 50 μm; (inset of U) 10 μm. Histological and immune-staining images are representative of 3 Transwell samples or animals examined. See also Figure S5.

**Figure S5:**
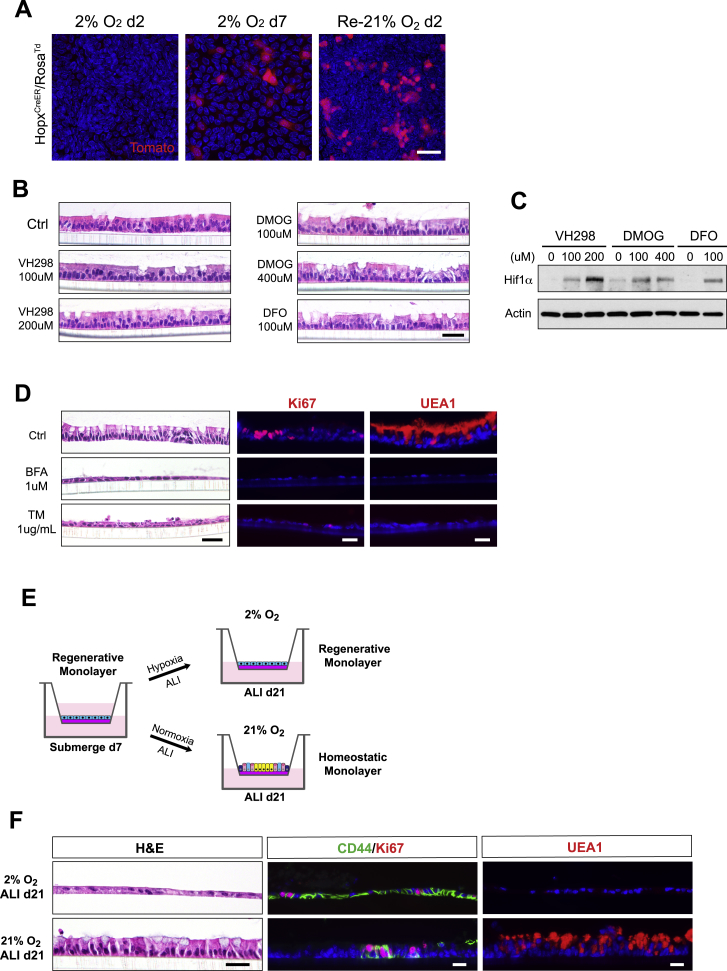
Oxygen Tension Functions as an Important Switch between Injury and Regeneration, Related to Figure 6 (A) Hopx expression was assayed following indicated time points after exposure to 2% O2 or re-exposure to 21% O2 by transient 4-OH tamoxifen labeling (24hrs prior) in ALI monolayers derived from *Hopx*^*CreER*^*/Rosa*^*Td*^ mice. (B-C) H&E stained sections of ALId21 monolayers treated with indicated concentrations of HIF activators including VH298, DMOG and DFO for 48hrs (B). Immunoblots for the Hif1α protein in cell lysates following 8hrs of the indicated HIF activator treatment (C). (D) H&E, Ki67 and UEA1 stained sections from ALId21 monolayers treated with ER stress inducers including BFA and TM for 48hrs. (E) Scheme for assessing the effect of low oxygen tension on the regenerative capacity of ALId0 cells. After 7 days of initial submersion, monolayer cells were subject to ALI in either 2% O_2_ or 21% O_2_ condition for 21 days. (F) ALId21 monolayers treated with either 2% O_2_ or 21% O_2_ were examined for cell morphology (left panels), Cd44/Ki67 double staining (mid panels) and UEA1 staining (right panels). Bars: (A, B, D and F) 25 μm. Histological and immuno-fluorescent images are representative of at least three samples examined.

An oxygen-depleted microenvironment was observed within colonic crypts during colitis-induced injury whereby infiltrating immune cells undergo a “respiratory burst” that rapidly consumes the locally available oxygen (Campbell et al., 2014). We asked if imposing this colitis-relevant condition on homeostatic monolayers could reproduce the epithelial flattening phenotype we observed with re-submersion and DSS-induced colitis. Indeed, switching the culturing condition of ALId21 cells to 2% O_2_ (Figure 6A) resulted in rapid flattening of the columnar epithelium, loss of goblet cells and a pause in cell proliferation within 48 h (Figures 6B–6P). We also observed a robust activation of UPR target genes shortly after switching to 2% O_2_ (Figure 6Q), along with statistically significant increases of regenerative markers (Figure 6R) similar to the re-submerged monolayer. Interestingly, Hopx transcription was transiently diminished after switching to 2% O_2_ and returned to baseline levels both after longer exposure and re-exposure to 21% O_2_ (Figure S5A). These data suggest that lowering oxygen tension can trigger a cellular injury-like status resembling features seen with both re-submerged monolayers and atrophic crypts.

To dissect which of the hypoxic stress pathways can confer the injury phenotype, we first tested if accumulation of HIF1α protein alone was sufficient to induce epithelial flattening. Treatment of ALId21 cells with multiple HIF activators including VH298, dimethyloxalylglycine (DMOG), and deferoxamine mesylate (DFO) individually did not result in morphological alterations of the monolayer (Figure S5B), despite the accumulation of Hif1α protein with treatment (Figure S5C). In contrast, treatment of ER stress inducers including brefeldin A (BFA) and tunicamycin (TM) caused epithelial flattening, loss of goblet cells, and decreased cell proliferation (Figure S5D), suggesting that ER stress can mediate the epithelial response to low oxygen tension.

To determine if a sufficient oxygen tension is required for the regenerative process powered by Hopx^+^ CARSCs *in vitro*, we cultured ALId0 cells under either 2% or 21% O_2_ with ALI for a period of 21 days (Figure S5E). As expected, cells cultured under normoxic conditions regenerated and developed both Ki67^+^ CD44^+^ crypt-like foci and differentiated lineages such as goblet cells (Figure S5F). In contrast, cells under the hypoxic condition maintained a CD44^+^ undifferentiated state with suppressed cell proliferation and showed no evidence of goblet cell differentiation (Figure S5F) even in the presence of ALI. Given the crucial role of oxygen tension in governing the switch between injury and regeneration in our *in vitro* model, we examined Hif1α protein levels in atrophic and hypertrophic crypts in DSS-induced colitis. Compared to controls not treated with DSS, the level of nuclear Hif1a protein was greatly enhanced in atrophic crypts at the injury phase and subsequently attenuated in the hypertrophic crypts during the regeneration stage (Figures 6S–6U). This dynamic alteration of oxygen tension was consistent with our *in vitro* model and suggested that oxygen tension may function as an important switch that controls the transformation between injury and regeneration for colonic crypts.

## Discussion

Colonic epithelial injury repair in response to damage caused by excessive inflammation remains a poorly understood process despite the importance of successful mucosal healing in achieving long-term remission for IBD patients (Baert et al., 2010, Henderson et al., 2011, Schnitzler et al., 2009). One major challenge has been a lack of *in vitro* model systems that recapitulate key aspects of epithelial injury and regeneration, in particular, modeling the regenerative stem cell population capable of re-establishing homeostasis following injury. In this study, we identified an *in vivo* stem cell population marked by Hopx expression that emerges from hypertrophic crypts present during recovery from colitis in both mice and humans. The Hopx^+^ CARSCs have diminished Lgr5 expression but are enriched for fetal biomarkers, suggestive of a primitive state. Lineage tracing and ablation experiments established that these cells could functionally contribute to epithelial regeneration during DSS-induced colitis. Prior to the emergence of Hopx^+^ CARSCs, atrophic crypts negative for both Lgr5 and Hopx prevailed in damaged areas concomitant with the injury phase. To model the atrophic, hypertrophic, and homeostatic crypts that occur during cycles of colonic epithelial injury and regeneration, we established a long-term colonic monolayer culture that contains regenerative stem cells resembling Hopx^+^ CARSCs. These *in vitro* Hopx^+^ CARSCs are capable of regenerating and forming a differentiated monolayer composed of numerous homeostasis-like “opened” crypts. Remarkably, the mature monolayer could be reverted to a Hopx^+^ regenerative state preceded by an ‘injury phase’ characterized by lack of proliferating cells, goblet cells and Hopx expression, similar to the atrophic crypts observed *in vivo* during colitis-induced injury. We demonstrate that oxygen tension functions as a switch for homeostasis-injury-regeneration cycle that can be reenacted in this *in vitro* model of colonic injury and repair.

The existence of a functional regenerative stem cell population has remained elusive for colitis-associated repair. Fetal-like reprograming of the regenerative epithelium has been described in mouse models following injury to the stomach and intestine (Fernandez Vallone et al., 2016, Nusse et al., 2018, Yui et al., 2018). However, whether these fetal-like cells are functionally required for re-establishing homeostasis has not been tested by lineage tracing or ablation experiments. The Hopx^+^ regenerative stem cells we observed in this study represent a transiently acquired cellular state during recovery and possess the ability to functionally contribute to the regenerative process in colitis. They co-express a number of fetal-like biomarkers and thus overlap phenotypically with the previously proposed fetal-like reprogrammed cells. Notably, these regenerative Hopx^+^ cells can likely be distinguished from the slow-cycling Hopx^+^ stem cells found in homeostasis because they exist in two different functional states, separated by a transient discontinuation of Hopx expression during the injury phase. Also, the regenerative Hopx^+^ stem cells are characterized by a rapid proliferation rate and co-expression of fetal-like markers during repair, neither of which are features of slow-cycling Hopx^+^ stem cells. Given these unique properties of this newly identified Hopx^+^ cell population, we have named it CARSC. Interestingly, Hopx^+^ alveolar epithelial cells have been implicated to play a role in pulmonary fibrosis (Ota et al., 2018), arguing for a unifying role for cells marked by Hopx. However, adult stem cells residing in organs of distinct developmental origins (e.g., lung and colon) can differ substantially in their overall expression profiles, behaviors, and functions even though they may share a subset of common markers.

While it is possible that the slow-cycling intestinal stem cells marked by Hopx in homeostasis can give rise to the CARSCs seen during colitis-associated regeneration, questions remain on the exact origin of these cells. Recent studies demonstrated that Atoh1^+^ secretory progenitors labeled in homeostasis can contribute to repair during DSS-induced colitis; ablating these cells negatively impact regeneration (Castillo-Azofeifa et al., 2019, Ishibashi et al., 2018, Tomic et al., 2018). Therefore, Atoh1^+^ progenitors may constitute a cell population that can exhibit cell plasticity and convert to Hopx^+^ CARSCs during repair. In addition, committed progenitors of other lineages including enteroendocrine cells and enterocytes may also be able to contribute to the emergence of Hopx^+^ CARSCs, given their known plasticities during intestinal repair (Buczacki et al., 2013, Jadhav et al., 2017, Tetteh et al., 2016, van Es et al., 2012, Westphalen et al., 2014, Yu et al., 2018).

The monolayer system we have developed here offers several unique advantages to aid the mechanistic study of epithelial injury and regeneration: First, unlike other *in vitro* methods that normally only model one specific cell fate in repair (Miyoshi et al., 2017, Yui et al., 2018), our system provides a platform for continuous manipulation of cell status over time. For example, it is unclear whether atrophic crypts that mark the injury phase of colitis can serve as a precursor for the hypertrophic crypts in recovery phase. Our *in vitro* data suggest that this level of cell plasticity could indeed occur. Second, in contrast to *in vivo* colitis models in which injured and regenerative crypts may be mixed with unaffected ones at a given time, our method allows the study of epithelial injury repair in a synchronized manner in both space and time. Third, our system permits the assessment of the direct impact of relevant stress signaling on epithelial cell fate decisions. Both hypoxic injury and ER stress commonly occur in IBD and mouse models of colitis (Giatromanolaki et al., 2003, Hatoum et al., 2005, Karhausen et al., 2004, Kaser et al., 2008, Shah et al., 2008, Shkoda et al., 2007). Hypoxia is known to regulate epithelial barrier function in colitis (Karhausen et al., 2004). Epithelial mutations in the UPR pathway can either lead to or influence colitis pathology (Barrett et al., 2008, Bertolotti et al., 2001, Brandl et al., 2009, Cao et al., 2013, Kaser et al., 2008, McGovern et al., 2010). Our *in vitro* model revealed that these stresses can directly induce features of epithelial injury (e.g., loss of proliferating and goblet cells) and may serve as a key switch for cell fate alterations during injury repair.

Finally, these long-lived, self-organizing 2D monolayers will have utility for defining host-microbe and epithelial-mesenchymal interactions that require experimental designs with sustained time frames. Previous studies reported self-organizing 2D monolayers derived from freshly isolated crypts (Gunasekara et al., 2018, Liu et al., 2018, Thorne et al., 2018). However, this experimental system is conducive only to short-term experiments; the resulting monolayers are often not continuous and the self-organizing phenotype could possibly be a result of direct crypt unfolding. One example of the utility of a complete, long-term 2D monolayer system described in our study is that the accessible apical surface supports the complete sexual growth of *Cryptosporidium parvum* (Wilke et al., 2019). In addition, other host cell types such as macrophages and fibroblasts play crucial roles in regulating responses to mucosal injury. Therefore, future efforts should be made to incorporate other cellular components into this epithelial system to allow better understanding of the intricate nature of mucosal repair.

## STAR★Methods

### Key Resources Table

**Table:** 

REAGENT or RESOURCE	SOURCE	IDENTIFIER
**Antibodies**		

Rat monoclonal anti-Ki67, eFluor 570 conjugated	ThermoFisher Scientific	Cat# 41-5698-82; RRID: AB_11220285
Rat monoclonal anti-Ki67, FITC conjugated	ThermoFisher Scientific	Cat# 11-5698-82; RRID: AB_11151330
Rabbit polyclonal anti-EpCAM	Abcam	Cat# ab71916; RRID: AB_1603782
Rat monoclonal anti-mouse EpCAM, BV421 conjugated	Biolegend	Cat# 118225; RRID: AB_2563983
Goat polyclonal anti-Tacstd2 (Trop2)	R&D systems	Cat# AF1122; RRID: AB_2205662
Rabbit monoclonal anti-Tacstd2 (Trop2)	Abcam	Cat# ab214488; RRID: AB_2811182
Mouse monoclonal anti-Villin	Thermo Fisher	Cat# MA5-12227; RRID: AB_10980388
Rabbit polyclonal anti-Slc26a3	Novus Biologicals	Cat# NBP1-84450; RRID: AB_11031898
Goat polyclonal anti-Muc2	Santa Cruz	Cat# sc-13312; RRID: AB_2146672
Rabbit polyclonal anti-Chga	Abcam	Cat# ab15160; RRID: AB_301704
Rabbit polyclonal anti-RFP	Rockland	Cat# 600-401-379; RRID: AB_2209751
Chicken polyclonal anti-GFP	Abcam	Cat# ab13970; RRID: AB_300798
Rat monoclonal anti-Ly6a (Sca-1), FITC conjugated	Biolegend	Cat# 122505; RRID:AB_756190
Rat monoclonal anti-Ly6g	Biolegend	Cat# 127601; RRID: AB_1089179
Rabbit polyclonal anti-Actin	MilliporeSigma	Cat# A2066; RRID: AB_476693
Rat monoclonal anti-CD44, Alexa Fluor 488 conjugated	Biolegend	Cat# 103015; RRID: AB_493678
Mouse monoclonal anti-β-Catenin	BD Biosciences	Cat# 610154; RRID: AB_397555
Mouse monoclonal anti-phospho-Histone H3 (Ser10)	Cell Signaling Technology	Cat# 9706; RRID: AB_331748
Rabbit monoclonal anti-Hif1α	Abcam	Cat# ab179483; RRID: AB_2732807
Rabbit monoclonal anti-phospho-Perk	Cell Signaling Technology	Cat# 3179; RRID: AB_2095853
Rabbit monoclonal anti-Perk	Cell Signaling Technology	Cat# 3192; RRID: AB_2095847
Rabbit polyclonal anti-phospho-eIF2α	Cell Signaling Technology	Cat# 9721; RRID: AB_330951
Rabbit polyclonal anti-eIF2α	Cell Signaling Technology	Cat# 9722; RRID: AB_2230924
Rabbit polyclonal anti-phospho-IRE1α	ThermoFisher Scientific	Cat# PA1-16927; RRID: AB_2262241
Rabbit monoclonal anti-IRE1α	Cell Signaling Technology	Cat# 3294; RRID: AB_823545
Rabbit polyclonal anti-Hspa5 (BiP)	Cell Signaling Technology	Cat# 3183; RRID: AB_10695864
Rabbit polyclonal anti-Ero1l	Cell Signaling Technology	Cat# 3264; RRID: AB_823684
Mouse monoclonal anti-β-Actin	Abcam	Cat# ab6276; RRID: AB_2223210

**Biological Samples**		

Formalin-fixed paraffin blocks of colonic resection/biopsy samples derived from patients with ulcerative colitis	Washington University in St. Louis Department of Pathology & Immunology	N/A

**Chemicals, Peptides, and Recombinant Proteins**		

Tamoxifen	MilliporeSigma	Cat# T5648
Diphtheria toxin	MilliporeSigma	Cat# D0564
Dextran Sodium Sulfate	TbD Consultancy	Cat# DB001-500 g
2,4,6-Trinitrobenzenesulfonic acid (TNBS)	MilliporeSigma	Cat# 2297-10ML
Paraformaldehyde (32%)	Electron Microscopy Sciences	Cat# 15714
Tissue-Tek Oct Compound	Fisher Scientific	Cat# 50-363-579
Agar	MilliporeSigma	Cat# A7921
Trilogy	MilliporeSigma	Cat# 920P
Rhodamine-labeled UEA I	Vector Laboratories	Cat# RL-1062; RRID: AB_2336769
Protease/phosphatase inhibitor cocktail (100x)	Cell Signaling Technology	Cat# 5872S
Blocking One buffer	Nacalai	Cat# 03953-95
Superblock (TBS) blocking buffer	ThermoFisher Scientific	Cat# 37537
DNase1	MilliporeSigma	Cat# 10104159001
Dispase 1	MilliporeSigma	Cat# 11097113001
Collagenase 1	ThermoFisher Scientific	Cat# 17100-017
Sytox-Red	Biolegend	Cat# 425301
Propidium Iodide	Biolegend	Cat# 421301
Brefeldin A (BFA)	MilliporeSigma	Cat# B7651-5MG
Tunicamycin (TM)	MilliporeSigma	Cat# T7765-5MG
VH298	Abcam	Cat# ab230370
Dimethyloxaloylglycine (DMOG)	MilliporeSigma	Cat# 89464-63-1
(Z)-4-Hydroxytamoxifen	MilliporeSigma	Cat# H7904
Y-27632	R&D Systems	Cat# 1254
Matrigel	Corning	Cat# 354234
Trypsin (10x)	MilliporeSigma	Cat# T4549

**Critical Commercial Assays**		

RNeasy Micro Kit	QIAGEN	Cat# 74004
iScript Reverse Transcription Supermix for RT-PCR	Bio-Rad	Cat# 1708841
TB Green Advantage qPCR Premix	Takara	Cat# 639676
VECTASTAIN Elite ABC HRP Kit	Vector Laboratories	Cat# PK-6100
DAB Peroxidase (HRP) Substrate Kit	Vector Laboratories	Cat# SK-4100
RNAscope Wash Buffer Reagents	ACDBio	Cat# 310091
RNAscope Target Retrieval Reagents	ACDBio	Cat# 322000
RNAscope H2O2 & Protease Plus	ACDBio	Cat# 2003382
RNAscope 2.5 HD Detection Reagents - Red	ACDBio	Cat# 2002657
Pierce BCA Protein Assay Kit	ThermoFisher Scientific	Cat# 23225
SuperSignal West Pico PLUS Chemiluminescent Substrate	ThermoFisher Scientific	Cat# 34580
SuperSignal West Femto Maximum Sensitivity Substrate	ThermoFisher Scientific	Cat# 34094
Click-iT EdU Cell Proliferation Kit for Imaging, Alexa Fluor 488 dye	ThermoFisher Scientific	Cat# C10337

**Deposited Data**		

Raw data files for RNA sequencing	This paper	GEO: GSE127172

**Experimental Models: Cell Lines**		

Mouse: L-WRN	Miyoshi and Stappenbeck, 2013; ATCC	Cat# CRL-3276
Mouse: C57BL/6J colonic spheroid line	This paper	N/A
Mouse: *Lgr5-EGFP-IRES-CreERT2/Rosa-LSL-tdTomato* colonic spheroid line	This paper	N/A
Mouse: *Hopx-CreERT2/Rosa-LSL-tdTomato* colonic spheroid line	This paper	N/A
Mouse: *Hopx-CreERT2/Rosa-LSL-DTR* colonic spheroid line	This paper	N/A
Mouse: *Hopx-GFP* colonic spheroid line	This paper	N/A
Mouse: *Axin2-CreERT2-tdTomato* colonic spheroid line	This paper	N/A

**Experimental Models: Organisms/Strains**		

Mouse: C57BL/6J	The Jackson Laboratory	Cat# 000664
Mouse: BALB/cJ	The Jackson Laboratory	Cat# 000651
Mouse: B6.129P2-Lgr5tm1(cre/ERT2)Cle/J (*Lgr5-EGFP-IRES-CreERT2*)	The Jackson Laboratory	Cat# 008875
Mouse: Hopxtm2.1(cre/ERT2)Joe/J (*Hopx-CreERT2*)	The Jackson Laboratory	Cat# 017606
Mouse: Hopxtm3.1Joe/J (*Hopx-GFP*)	The Jackson Laboratory	Cat# 029271
Mouse: B6.Cg-Gt(ROSA)26Sortm9(CAG-tdTomato)Hze/J (*Rosa-LSL-tdTomato*)	The Jackson Laboratory	Cat# 007909
Mouse: C57BL/6-Gt(ROSA)26Sortm1(HBEGF)Awai/J (*Rosa-LSL-DTR*)	The Jackson Laboratory	Cat# 007900
Mouse: Axin2tm1.1(cre/ERT2/tdTomato)Smr (*Axin2-CreERT2-tdTomato*)	Gift of E.E. Morrisey, University of Pennsylvania, Philadelphia, PA; Frank et al., 2016	N/A

**Oligonucleotides**		

qPCR primers, see also Table S7	This paper	N/A
RNAscope Probe-Mm-Lgr5	ACDBio	Cat# 312171
RNAscope Probe-Mm-Hopx	ACDBio	Cat# 405161
RNAscope Probe-Hs-LGR5	ACDBio	Cat# 311021
RNAscope Probe-Hs-HOPX	ACDBio	Cat# 423001

**Software and Algorithms**		

GSEA	Broad Institute; Subramanian et al., 2005	http://software.broadinstitute.org/gsea/index.jsp
Enrichr	Ma’ayan Laboratory, Icahn School of Medicine at Mount Sinai, New York, NY; Chen et al., 2013, Kuleshov et al., 2016	https://amp.pharm.mssm.edu/Enrichr/
EdgeR	Bioconductor	https://bioconductor.org/packages/release/bioc/html/edgeR.html
Limma	Bioconductor	http://bioconductor.org/packages/release/bioc/html/limma.html
GAGE	Bioconductor	https://bioconductor.org/packages/release/bioc/html/gage.html
ImageJ	NIH	https://imagej.nih.gov/ij/
Graphpad Prism 8	Graphpad	https://www.graphpad.com/scientific-software/prism/

### Lead Contact and Materials Availability

Further information and requests for reagents may be directed to and will be fulfilled by the Lead Contact Thaddeus S. Stappenbeck (stappenb@wustl.edu).

New materials generated in this study, including colonic spheroid lines derived from specific mouse genetic lines, are available from the Lead Contact without restrictions.

### Experimental Model and Subject Details

#### Mice

The *Lgr5-EGFP-IRES-CreERT2*, *Hopx-CreERT2*, *Hopx-GFP, Rosa-LSL-DTR* and *Rosa-LSL-tdTomato* mice were purchased from Jackson Laboratory. The *Axin2-CreERT2-tdTomato* line was a kind gift from Dr. Edward E. Morrisey (University of Pennsylvania). Male mice 8-12 weeks of age were used to perform experiments. Detailed information on generation and genotyping of those mouse lines has been published previously (Barker et al., 2007, Buch et al., 2005, Frank et al., 2016, Takeda et al., 2011, Takeda et al., 2013). All animal work was performed in accordance to the protocols approved by the Washington University School of Medicine Animal Studies Committee.

#### Human Tissue

Seven pairs of retrospective formalin-fixed paraffin blocks containing inflamed and uninflamed regions of colonic surgical resection or biopsy samples from subjects with ulcerative colitis were obtained from Department of Pathology and Immunology at Washington University in St. Louis. Five μm sections from these paraffin blocks were stained for localization of TACSTD2 protein using a rabbit monoclonal antibody (Abcam) by IHC or Hopx and Msln mRNA by RNAscope-based i*n situ hybridization*. This study was approved by the Washington University Institutional Review Board (IRB #201209047).

#### Mouse colonic spheroid lines

Mouse colonic stem cell spheroid lines were derived directly from 8-week-old male mice with indicated genotypes as previously described (Miyoshi and Stappenbeck, 2013). Briefly, 1cm segment of distal colon was minced and digested in 2mg/mL Collagenase I solution (ThermoFisher Scientific) for 30-40min, with vigorous pipetting every 10min. The crypt compartment was collected by centrifugation, washed twice with DMEM/F12 media (MilliporeSigma), resuspended in 15μL cold Matrigel and plated onto a 24-well tissue culture plate with supplementation of 50% L-WRN conditioned medium.

### Methods Details

#### ALI culture experiments

Adult mouse colonic spheroids were maintained and passaged up to 30 generations in 50% L-WRN conditioned medium as previously described (Miyoshi and Stappenbeck, 2013). Spheroids were then dissociated into single cells by 0.25% trypsin (MilliporeSigma) digestion and seeded onto Transwells (Corning) pre-coated by 10% Matrigel (Corning) for 20min. ∼20,000 cells were seeded per well. Initially, cells were submerged in 150μL 50% L-WRN media with 10uM Rock inhibitor Y-27632 (R&D Systems). Medium in the upper chamber of the Transwell was removed after 7 days to create an air-liquid interface (ALI). Cells were grown in ALI with 50% L-WRN medium in the bottom chamber for additional 14-28 days to reach maturity (changing the medium every three days). To re-submerge the ALI culture, 150μL 50% L-WRN medium was added into the upper chamber. Medium in the upper chamber was removed after 7 days to subject cells to another round of ALI mediated differentiation. To study the effect of hypoxia on ALI cells, monolayers of cells on Transwells were incubated under an atmosphere of 2% O_2_, 5% CO2 and 93% nitrogen at 37°C for up to 48 h, with medium only present in the bottom chamber. For transient labeling or lineage tracing experiments, ALI cultures were treated with 500nM-1μM 4OH-tamoxifen (MilliporeSigma) in 50% L-WRN medium for 12 h followed by replacing with 50% L-WRN medium alone. tdTomato fluorescence was monitored 1 day later for transiently labeling experiments and 3 weeks later for lineage tracing experiments. For treatment with HIF activators, 100μM and 200μM VH298 (Abcam), 100μM and 400μM dimethyloxaloylglycine (DMOG, MilliporeSigma), or 100μM Deferoxamine mesylate (DFO, Abcam) was individually applied to the bottom compartment of ALId21 monolayers for 48hrs. For treatment with ER stress inducers, 1μM brefeldin A (BFA, MilliporeSigma) or 1μg/mL tunicamycin (TM, MilliporeSigma) was applied to the bottom compartment of ALId21 monolayers for 48hrs. For EdU pulse-chase experiments, ALId14 monolayers were treated with 10μM EdU for 3hrs, replaced with media without EdU, and followed for various amount of time before using the EdU Click-it kit (Thermofisher Scientific) to detect EdU signals. Ki67 co-staining was performed to identify proliferative zones in the monolayers.

#### DSS-induced Colitis

Male C57BL/6J mice of 8-12 weeks of age were treated for 7 days with drinking water supplemented with 2.5% Dextran Sulfate Sodium (DSS) (TbD Consultancy) before switching to unsupplemented (‘regular’) drinking water. Mice were sacrificed at various time points during DSS treatment or the water recovery phase.

#### Acute TNBS-induced Colitis

Male BALB/c mice of 8-12 weeks of age were treated with 2.5% 2,4,6-Trinitrobenzenesulfonic acid (TNBS, MilliporeSigma) in 50% ethanol by enema and sacrificed at various time points after treatment for histologic analysis in distal colons.

#### Lineage Tracing and Cell Ablation

*Hopx*^*CreER*^*/Rosa*^*Td*^ and *Lgr5*^*CreER*^*/Rosa*^*Td*^ mice were treated with 2.5% DSS for 7 days and switched to regular drinking water afterward. Tamoxifen was dissolved in corn oil at 20mg/mL and delivered to mice at 0.1g/kg weight through intraperitoneal injection on designated days. For ablation experiments at the regenerative stage, *Hopx*^*CreER*^*/Rosa*^DTR^ mice and their WT littermate controls were treated with DSS in the drinking water and supplied with tamoxifen chow 7 days after switching to regular drinking water. Three doses of diphtheria toxin (DT 50μg/kg; MilliporeSigma) were injected intraperitoneally into mice every other day, beginning 14 days after switching to regular water. Mice were sacrificed 2 days after the last dose of diphtheria toxin. For ablation during homeostasis, *Hopx*^*CreER*^*/Rosa*^DTR^ mice and their WT littermate controls were injected with tamoxifen on day 1, 3, and 5, DT on day 2, 4, and 6 and sacrificed 2 days after the last DT injection. For ablation during injury, *Hopx*^*CreER*^*/Rosa*^DTR^ mice and their WT littermate controls were treated with DSS for 7 days followed by a water washout period (no DSS), with a total of 3 doses of tamoxifen and DT administration every other day starting from DSS day 6. Mice were sacrificed 2 days after last DT injection.

#### Tissue Preparation and Histology

Dissected full-length colons were pinned out and fixed in 4% paraformaldehyde (PFA) for 16 h at 4°C. The fixed tissue was washed in phosphate buffered saline (PBS) three times before dehydration in 70% ethanol. Tissues were embedded in 2% agar followed by paraffin embedding, sectioning, hematoxylin and eosin staining, and alcian blue staining. ALI cultured cells were fixed in 4% PFA for 30min at room temperature and treated similarly to the tissues for histological examinations.

#### Immunostaining

Slides were de-paraffinized and subjected to heat-induced antigen retrieval in Trilogy (MilliporeSigma) solution for 20min. Slides were blocked in PBS containing 2% BSA, 5% serum and 0.1% Triton-X for 1hr at room temperature, and then treated with primary antibodies at desired dilutions for 16 h at 4°C. Slides were then washed 3 times in PBS and incubated with secondary antibodies conjugated with fluorophores (Thermo Fisher Scientific) for 1 h at room temperature. For immunohistochemistry, VECTASTAIN Elite ABC HRP Kit and I DAB Peroxidase (HRP) Substrate Kit (Vector Laboratories) were used to develop colors. For whole mount staining with ALI culture, cells were fixed in 4% PFA for 30 min at room temperature and washed in PBS to remove the mucus layer. Cells were then blocked, incubated with primary antibodies and followed the same procedures used on slide sections. Fluorescent images were acquired with a Zeiss Axiovert 200M inverted microscope. Bright field histological and RNA *in situ* hybridization images were acquired with an Olympus BX51 microscope. Adobe Photoshop was used to uniformly adjust brightness and contrast. ImageJ was used to quantify cell height. Information about primary antibodies and dilutions used in the assay can be found in “Key Resources Table.”

#### RNAscope *in situ* hybridization

Tissues were fixed in 4% PFA for 16 h at 4°C and balanced in 20% sucrose/PBS for at least 8 h before embedded in OCT compound (Fisher Scientific). Five micron-thick cryosections were used for RNAscope based *in situ* hybridization according to protocols recommended by the manufacturer (ACDBio). See “Key Resources Table” for details about probes used in the assays.

#### Quantitative RT-PCR

Total RNA was isolated from ALI cultures by using RNeasy micro kit (QIAGEN). 500ng of RNA was used for cDNA synthesis by iScript Reverse Transcription Supermix Kit (Bio-Rad). qPCR was performed using TB Green Advantage qPCR Premix (Takara). Due to significant changes in glycolysis documented in these experiments, Sdha or Hsp90ab was used for housekeeping genes for normalization instead of Gapdh. See also Table S7 for details about primers used for qPCR.

#### RNA-Seq

Total RNA quality was tested by Bioanalyzer. The RNA-Ribo-Zero method was employed for cDNA library construction. Sequencing datasets (produced using an Illumina HiSeq3000 instruments were analyzed as follows. Reads were aligned to the reference mouse genome using STAR software. Gene counts were derived from uniquely aligned reads. To identify differentially expressed genes, TMM normalization factors were calculated using EdgeR to adjust for differences in sample read numbers. Differential gene expression analysis was performed in Limma with voom. Gene Ontology (GO) and KEGG pathways significantly altered in sample groups were identified by using GAGE. Differentially expressed gene lists were also analyzed by using Enrichr (Chen et al., 2013, Kuleshov et al., 2016). To compare the similarities of RNA profiles between our data and published data, custom “gene sets” were generated from the top 300-500 upregulated genes from previous studies on goblet cell, enteroendocrine cell, DSS-induced regenerative epithelium and fetal spheroids (Jadhav et al., 2017, Mustata et al., 2013, Yui et al., 2018). Functional enrichment of these two gene sets in the ALId21, ALId0 or Re-submerge d7 transcriptomes was tested using the GSEA software package (Subramanian et al., 2005).

#### Immunoblotting

ALI cultured cells were first lysed in Lammaeli sample buffer supplemented with protease/phosphatase inhibitor cocktail (Cell Signaling). Protein concentration was quantified by the Pierce BCA Protein Assay Kit (Thermo Fisher Scientific). 10μg of denatured protein per sample was loaded onto Any kD Mini-PROTEAN® TGX Precast Protein Gels (Bio-Rad) for SDS-PAGE, and then transferred to nitrocellulose membranes using a Trans-Blot SD. Semi-Dry transfer cell (Bio-Rad). Membranes were blocked in either Blocking One buffer (Nacalai) for non-phosphorylated protein targets or Superblock buffer (Thermo Fisher Scientific) for phosphorylated targets for 1 h at room temperature, and then incubated with primary antibodies (1:1000 dilution) for 16 h at 4°C. Membranes were washed with Tris buffered saline containing 0.1% Tween-20 (TBST) 4 times (10minutes/cycle) with sufficient rocking, and then incubated with poly-horseradish peroxidase conjugated secondary antibodies (Abcam, 1:15000) for 1 h at room temperature. Membranes were subjected to four more cycles of washing in TBST before using the SuperSignal West Pico PLUS Chemiluminescent Substrate or SuperSignal West Femto Maximum Sensitivity Substrate (ThermoFisher Scientific) to detect signals. Information about the primary antibodies used can be found in “Key Resources Table.”

#### LC-QTOF Mass spectrometry

Lactate was extracted in ice-cold methanol (400μL) by vortex mixing. Following centrifugation (8,000 x g at 4°C), a 200μL aliquot of the resulting supernatant was evaporated to dryness. Dried samples were then re-suspended in 100 μL of 10% methanol. The analysis was done on an Agilent 1290 LC system coupled to an Agilent 6545 Q-TOF mass spectrometer (Santa Clara, CA) under negative ion mode. Five μL of prepared sample were injected onto a Scherzo SW-C18 column (2 × 100 mm, 3 μm, Imtakt, Portland, OR), which was heated to 35°C. The mobile phase consisted of 10mM ammonium formate in water (A) and 10mM ammonium formate in 90:10 acetonitrile/water (B). The flow rate was at 0.4 ml/min. The following gradient program was performed: from 0 to 10 min, mobile phase B eluted from 0% to 50% and then was kept for 3 min at 50% of B.

#### Flow cytometry

ALI cultured cells derived from WT, Hopx^GFP^ and Lgr5^GFP^ mouse strains (Barker et al., 2007, Takeda et al., 2013) were dissociated into single cells by 0.25% Trypsin and stained with Propidium Iodide (Biolegend) before sent for flow cytometry analysis (Becton Dickinson FACScan). Live single cells were gated to assess for GFP signal in these three lines. WT cells served as a negative control for gating GFP channel.

#### Single cell spheroid formation assay

*Hopx*^*CreER*^*/Rosa*^Td^ mice were treated with 2.5% DSS in drinking water for 7 days before switch to regular water. Hopx-tdTomato+ cells were labeled by a single injection of tamoxifen 13 days after the cessation of DSS treatment. Twenty-four hours after tamoxifen injection, the most distal 1cm section of colon was removed, opened, and treated with 30mM EDTA for 20minutes at 37°C followed by vigorous pipetting to enrich for crypts in the suspension. Single cell dissociation was then performed using an enzymatic cocktail containing 0.2mg/mL DNase1 (MilliporeSigma), 5mg/mL Dispase 1 (MilliporeSigma) and 2mg/mL Collagenase I (ThermoFisher Scientific) in the 37°C shaker at 250rpm. The cell suspension was incubated with EpCAM-BV421 (Biolegend) and Sytox-Red (Biolegend), which was used to gate for live epithelial cells during sorting (MoFlo). Sorted tdTomato+ epithelial cells were plated in Matrigel supplemented with 50% L-WRN medium and cultured for 6-7 days to count for spheroid formation efficiency.

#### Transmission electron microscopy

Cells were fixed with 2% paraformaldehyde/2.5% glutaraldehyde in 100mM cacodylate buffer, pH 7.2 for 1 h at 24°C. Ninety-five nanometer thick sections were cut with a Leica Ultracut UCT ultramicrotome, stained with uranyl acetate and lead citrate, and imaged on a JEOL 1200 EX transmission electron microscope (JEOL USA). Adobe Photoshop was used to uniformly adjust brightness and contrast. ImageJ was used to quantify the microvilli lengths.

### Quantification and Statistical Analysis

Statistical parameters including the exact value of n, precision measures (mean ± SD) and statistical method and significance are reported in the Results and Figure Legends. Data are judged to be statistically significant when p < 0.05. In figures, asterisks denote statistical significance: ^∗^, p < 0.05; ^∗∗^, p < 0.01.

### Data and Code Availability

The accession number for the RNA sequencing data reported in this study is NCBI GEO: GSE127172.

Software used for statistical analysis was Graphpad Prism v8.
Software used for Gene Set Enrichment Analysis is GSEA v3.0.
Software used for RNA-seq data pathway analysis is Enrichr (https://amp.pharm.mssm.edu/Enrichr/).
Software used for image processing is ImageJ v1.8.0 (https://imagej.nih.gov/ij/).
The R packages used to analyze RNA-seq data in this study are: EdgeR (https://bioconductor.org/packages/release/bioc/html/edgeR.html), Limma (http://bioconductor.org/packages/release/bioc/html/limma.html) and GAGE (https://bioconductor.org/packages/release/bioc/html/gage.html).

This study did not generate original code.

## Supplemental Information

Table S1. Gene Set for Goblet Cell Signature Tested in ALId21 versus ALId0, Related to Figure 2

Table S2. Gene Set for Enteroendocrine Cell Signature Tested in ALId21 versus ALI0, Related to Figure 2

Table S3. Gene Set for DSS-Associated Regenerative Epithelial Signature Tested in ALId0 versus ALId21, Related to Figure 3

Table S4. Gene Set for Fetal Epithelial Signature Tested in ALId0 versus ALId21, Related to Figure 3

Table S5. Gene Set for DSS-Associated Regenerative Epithelial Signature Tested in Re-Sub d7 versus ALId21, Related to Figure 4

Table S6. Gene Set for Fetal Epithelial Signature Tested in Re-Sub d7 versus ALId21, Related to Figure 4

Table S7 QPCR Primers, Related to STAR Methods
