# Supplementary material for: Long-Term Culture Captures Injury-Repair Cycles of Colonic Stem Cells
Source: Cell. 2019 Nov 14;179(5):1144–1159.e15. doi: 10.1016/j.cell.2019.10.015 (PMC6904908; doi:10.1016/j.cell.2019.10.015)
Supplement: Table S7 QPCR Primers, Related to STAR Methods [file mmc7.pdf]

Table S7 QPCR Primers, Related to STAR methods

Anxa1-F	TCTGACCAGCAGGAGCTTTC
Anxa1-R	TGGCTTCGTACAGCTTCTCG
Anxa8-F	AGCGGATTCTGGTGTGTCTC
Anxa8-R	TCCCATAATCTTCTCACCCGC
Ly6a-F	AGCTCCAAGGTGGGAGTAGT
Ly6a-R	AGGGTCATGAGCAGCAATCC
Ly6g-F	GAGAGGAAGTTTTATCTGTGCAGC
Ly6g-R	TCTCAGGTGGGACCCCAATA
Tacstd2-F	TCACCAAACGGAGGAAGTCG
Tacstd2-R	GAAGTCGGGGCATCCTACAG
Atf3-F	AGAGCTGAGATTCGCCATCC
Atf3-R	TTGTTTCGACACTTGGCAGC
Ero1L-F	GAATGTGAGCAAGCTGAGCG
Ero1L-R	CATACTCAGCATCGGGGGAC
Gadd34-F	CACGATCGCTTTTGGCAACC
Gadd34-R	TAGAAGTTGTGGGCGTCCCT
Hsp90ab1-F	GGCTATCCCATCACCCTCTATTT
Hsp90ab1-R	GGCTTCTCCTCATCCTCCTTATC
Sdha-F	GGTGAGAACAAGAAGGCATCAG
Sdha-R	GCCTACAACCACAGCATCAAA
Bnip3-F	GCTTTGGCGAGAAAAACAGC
Bnip3-R	ATGCTGAGAGTAGCTGTGCG
Ddit3-F	CCACCACACCTGAAAGCAGAA
Ddit3-R	AGGTGAAAGGCAGGGACTCA
Ddit4-F	CTTGTCCGCAATCTTCGCTG
Ddit4-R	TGGCTGCTAAGGTCCGAATG
Slc2a1-F	CGGGGTCTTAAGTGCGTCA
Slc2a1-R	ATAGCCGAACTGCAGTGATCC
sXbp1-F	CTGAGTCCGCAGCAGGTG
sXbp1-R	GTCCATGGGAAGATGTTCTGG
Vegfa-F	CACAGCAGATGTGAATGCAG
Vegfa-R	TTTACACGTCTGCGGATCTT
P4hb-F	GACTCAAGCGAAGTGACGGT
P4hb-R	TCAAAGTTGTTGCGGCCTTC
